# Co‐Benefits of Nutrition Interventions: A Systematic Review

**DOI:** 10.1111/obr.70159

**Published:** 2026-05-11

**Authors:** Phuong Nguyen, Siti Khadijah binti Mohamad Asfia, Vicki Brown, Nicole Ward, Moosa AlSubhi, Marufa Sultana, Lennert Veerman, Andrew Milat, Belen Zapata‐Diomedi, Jaithri Ananthapavan

**Affiliations:** ^1^ Deakin Health Economics, Institute for Health Transformation, School of Health and Social Development, Faculty of Health Deakin University Burwood Victoria Australia; ^2^ Global Centre for Preventive Health and Nutrition, Institute for Health Transformation, School of Health and Social Development, Faculty of Health Deakin University Burwood Victoria Australia; ^3^ Ministry of Women, Early Childhood and Community Wellbeing Development Kuching Sarawak Malaysia; ^4^ Public Health & Economics Modelling Group, School of Medicine and Dentistry Griffith University Gold Coast Australia; ^5^ Prevention Research Collaboration The University of Sydney Sydney Australia; ^6^ Centre for Urban Research RMIT University Melbourne Australia

**Keywords:** co‐benefits, diet, nutrition, obesity, outcome, prevention

## Abstract

**Introduction:**

Nutrition interventions aim to improve dietary quality and enhance health. They can also result in broader co‐benefits, including social, educational, and environmental outcomes. However, these co‐benefits remain under‐quantified. This review examined quantified non‐health outcomes of nutrition‐related policies, programs, and interventions.

**Methods:**

Following PRISMA guidelines, five databases (Medline Complete, Global Health, Environment Complete, SocINDEX, and Embase) were searched for studies published between January 2012 and September 2025. Search terms targeted co‐benefits and nutrition or food systems initiatives with a nutrition component. Non‐health outcomes were categorized into social, educational, and environmental domains. Risk of bias was assessed using the Mixed Methods Appraisal Tool.

**Results:**

Twenty studies evaluating 15 interventions were included. Educational co‐benefits were most commonly reported (*n* = 10), followed by social (*n* = 8) and environmental (*n* = 4). Three studies reported co‐benefits across multiple domains. Multicomponent interventions consistently showed positive educational and social impacts, though diverse measurement tools limited comparability. Environmental outcomes, notably reductions in greenhouse gas emissions, were consistently significant.

**Conclusion:**

Quantifying co‐benefits highlights the broader societal value of nutrition initiatives. Standardized outcome measures are essential to advance understanding of these impacts and inform comprehensive policy development.

**Abbreviations**
Cis: confidence intervals
CO_2_
: carbon dioxide
DALYs: disability‐adjusted life years
GHGE: greenhouse gas emissions
MMAT: Mixed Methods Appraisal Tool
NO_2_
: nitrogen dioxide
OMI: outcome measurement instrument
PA: physical activity
PO_4_
: phosphate
RCTs: randomized controlled trials
SD: standard deviation
SE: standard error
SFP: school breakfast program
SO_2_
: sulfur dioxide
SWiM: Synthesis without meta‐analysis
UK: United Kingdom
USA: United States of America

## 1 Introduction

An unhealthy diet is recognized as a major contributing factor to non‐communicable diseases, including obesity, cardiovascular disease, and cancer [1, 2, 3]. Diet quality globally is suboptimal, characterized by insufficient fruits, vegetables, and whole grains and excess sodium and sugar‐sweetened beverages, with only modest improvements since the 1990s [4]. The most recent GBD 2023 study shows that among the 10 leading risk factors for disease burden, high blood sugar and high body mass index (BMI) are increasing globally, while most other major risk factors have declined between 2010 and 2023 [5].

Nutrition interventions including taxes and subsidies, marketing regulations, improved food availability, and healthier retail environments have been widely recognized as essential components in the prevention of non‐communicable diseases [6, 7]. They include strategies from individual behavior change (such as dietary counseling and education) [8] to population‐level policy, systems, and environmental approaches [9]. It is increasingly apparent that nutrition interventions can yield broader non‐health benefits (co‐benefits) beyond diet quality and chronic‐disease prevention [10, 11]. These co‐benefits include economic gains (e.g., improved workforce productivity or reduced healthcare expenditures) [12, 13], environmental sustainability (such as lower greenhouse‐gas emissions and reduced food waste) [12, 14], social outcomes (for instance, enhanced community engagement or reduced social isolation) [12], and educational gains (e.g., improved academic performance or attitude) [12]. Recent reviews highlight that diets optimal for both health and planetary sustainability offer quantifiable environmental gains [15].

Research on co‐benefits of nutrition interventions has gained increasing attention in recent years. An umbrella review in 2021 summarized evidence across interventions targeting multiple risk factors, including unhealthy diet and obesity, and identified additional social (e.g., reduced inequity), economic (e.g., lower hospitalisations), and mental health (e.g., improved emotion regulation) benefits [12]. However, this review was limited to qualitative findings and did not quantify the range or magnitude of co‐benefits. Another more recent review reports that multicomponent policies such as labeling, reformulation and fiscal measures show double‐duty potential across nutrition and environmental outcomes [14].

Although some studies have examined individual domains of co‐benefits, there remains a lack of systematic synthesis that considers the multiple co‐benefits of nutrition interventions simultaneously. Previous reviews were limited to secondary analyses of systematic reviews that may have overlooked relevant primary studies. Quantifying and valuing co‐benefits can strengthen the investment case for prevention by providing a more holistic estimate of the societal value of nutrition interventions [10, 11]. Therefore, this review aims to identify and synthesize primary studies that quantify the social, educational, and environmental co‐benefits of nutrition‐ and diet‐related interventions.

## 2 Methods

### 2.1 Search Strategy

A systematic review was conducted following the Preferred Reporting Items for Systematic Reviews and Meta‐Analyses (PRISMA) guidelines [16] (PROSPERO registration number CRD42022358139). Five databases (Medline Complete, Global Health, Environment Complete, SocINDEX, and Embase) were searched for studies published between January 2012 and 13 September 2022. An updated search was conducted for all five original databases covering January 2022 to 30 September 2025. We also added one new database (CINAHL) and ran the search from 2012 to 30 September 2025. Three concepts and their related terms were applied across all databases: (1) co‐benefits: educational, social, and environmental; (2) type of intervention: programs, policy, and so on; and (3) intervention component: nutrition, healthy diet, multicomponent intervention, that is, nutrition with another component, or food system interventions with a nutrition component. Details of the search strategy are reported in the Supporting Information.

### 2.2 Inclusion and Exclusion Criteria

Health promoting nutrition interventions and obesity prevention interventions, that is, nutrition intervention with or without a physical activity (PA) component, were included. A nutrition intervention to prevent overweight and obesity or chronic diseases can be defined as a coordinated policy, program, or initiative designed to improve dietary behaviors, food environments, or population‐level access to healthy foods, with the goal of improving nutrition and health outcomes. These interventions may include actions such as school food programs (free school breakfast or healthy canteen menu), fiscal measures (e.g., subsidies or taxes), food environment/system modifications (food labeling), or community‐based initiatives that promote healthy eating [17]. We do not include micro‐nutrition intervention, for example, programs that supply vitamins to children at school.

No restrictions were placed on location or setting. Studies were excluded if they did not include a nutrition‐related intervention component or if the target population was characterized by specific health conditions such as overweight or obesity, cardiovascular disease, type 2 diabetes, cancer, or a population group that required specific dietary requirements such as athletes.

Studies such as randomized controlled trials (RCTs), cohort studies (with or without control), pre‐post studies, modeling studies, and observational studies were included. Quantitative and mixed methods studies (with quantified co‐benefits) were also included. Inclusion was restricted to studies published in the English language. Excluded studies consisted of qualitative studies, conference abstracts, commentaries, theses, protocols, and systematic reviews.

Outcomes of interest were co‐benefits of nutrition interventions, such as educational, social, and environmental impacts. Studies that only reported health outcomes (e.g., body mass index and dietary changes) or non‐quantified co‐benefits were excluded. Educational co‐benefit consists of both academic performance and academic behavior. Academic performance referred to general grade scores, reading tests, or test results for Mathematics, while academic behavior referred to school attendance, perceived academic self‐efficacy, student retention, suspensions, expulsions, written complaints about students, and tardiness to class. Social co‐benefits refer to an individual's social participation/connection, feelings of social isolation and loneliness, and perception of facilities in terms of safety and condition of neighborhood. For this study, we focus on the environmental co‐benefits as referring to the impact of the intervention on environmental sustainability outcomes such as greenhouse gas emissions (GHGE) (e.g., carbon dioxide CO_2_, sulfur dioxide SO_2_, and nitrogen dioxide NO_2_).

### 2.3 Data Extraction and Synthesis

Studies identified in the search were exported to EndNote 20 [18] for removal of duplicates and uploaded onto Covidence [19]. Independent double screening (by S.M., P.N., M.A., M.T., N.W., and M.S.) of title and abstract followed by full text was conducted. Conflicts were resolved by discussion between reviewers and additional reviewers (J.A. and V.B.) until consensus was reached at all stages of screening.

Study characteristics for the included studies were extracted by six reviewers (S.M., P.N., M.A., M.S., J.A., and N.W.) and checked by two reviewers (S.M. and P.N.). Data were extracted using an Excel template and included (where available): author(s), year of publication, study design, population, number of participants, participant demographics, intervention components, comparator, setting, country, health outcomes, quantified outcomes, and measurement methods for co‐benefits.

We utilized the Synthesis without meta‐analysis (SWiM) guideline to conduct a narrative synthesis (see Supporting Information) [20]. Outcomes were grouped into social, educational, and environmental impacts and presented in tables with descriptions of the setting, population (including age group or mean age), study design, and duration of study. Where applicable, the significance of the outcomes was reported with uncertainties around point estimates (*p* value, standard deviation [SD], standard error [SE], and confidence intervals [CIs]). The quantified co‐benefits were categorized into four themes: positive (statistically significant improvement after intervention), no impact (non‐significant improvement after intervention), mixed results (positive impact for participant/population sub‐groups but no impact on other groups, with or without any non‐significant negative impacts), and negative impact (statistically significant decline in outcomes). We initially intended to combine the quantified co‐benefits using a random‐effects meta‐analysis as per our research protocol. However, due to the heterogeneity in the outcomes reported across studies and diverse interventions, we were unable to estimate a pooled effect for the included co‐benefits.

### 2.4 Quality Assessment

Studies were assessed for risk of bias using the Mixed Methods Appraisal Tool (MMAT) [21] by one reviewer and checked for accuracy by another reviewer (S.M., P.N., M.A., M.S., and NW). The MMAT tool can be used to assess study quality for a range of study designs, including randomized controlled trials, non‐randomized studies, and quantitative descriptive studies. MMAT is reported to be an efficient and reliable tool for reviewing methodological quality [22]. The MMAT has two screening questions and five criteria suited to each of the study designs. Reviewers respond to each item question as “Yes,” “No,” or “Cannot tell” [21]. The outcome of the quality assessment was reported as the number of criteria fulfilled out of seven criteria, and studies were ranked from the greatest number of criteria fulfilled to the least. We categorized the study as high quality if more than 80% of the criteria were met, moderate if 60%–70% of the criteria were met, and low quality if less than 60% of the criteria were met [21].

## 3 Results

### 3.1 Overall Characteristic of Included Studies

Twenty out of 27,294 screened articles were included in the review (see Figure 1) [23, 24, 25, 26, 27, 28, 29, 30, 31, 32, 33, 34, 35, 36, 37, 38, 39, 40, 41, 42]. The majority of included studies (*n* = 13, 65%) were based in the United States of America (USA) [23, 24, 25, 28, 29, 30, 31, 32, 33, 34, 35, 36, 37], followed by three studies in the United Kingdom (UK) [38, 41, 42], and one study each based in China [39], France [40], Ethiopia [26], Australia [43], and Egypt [27].

**FIGURE 1:**
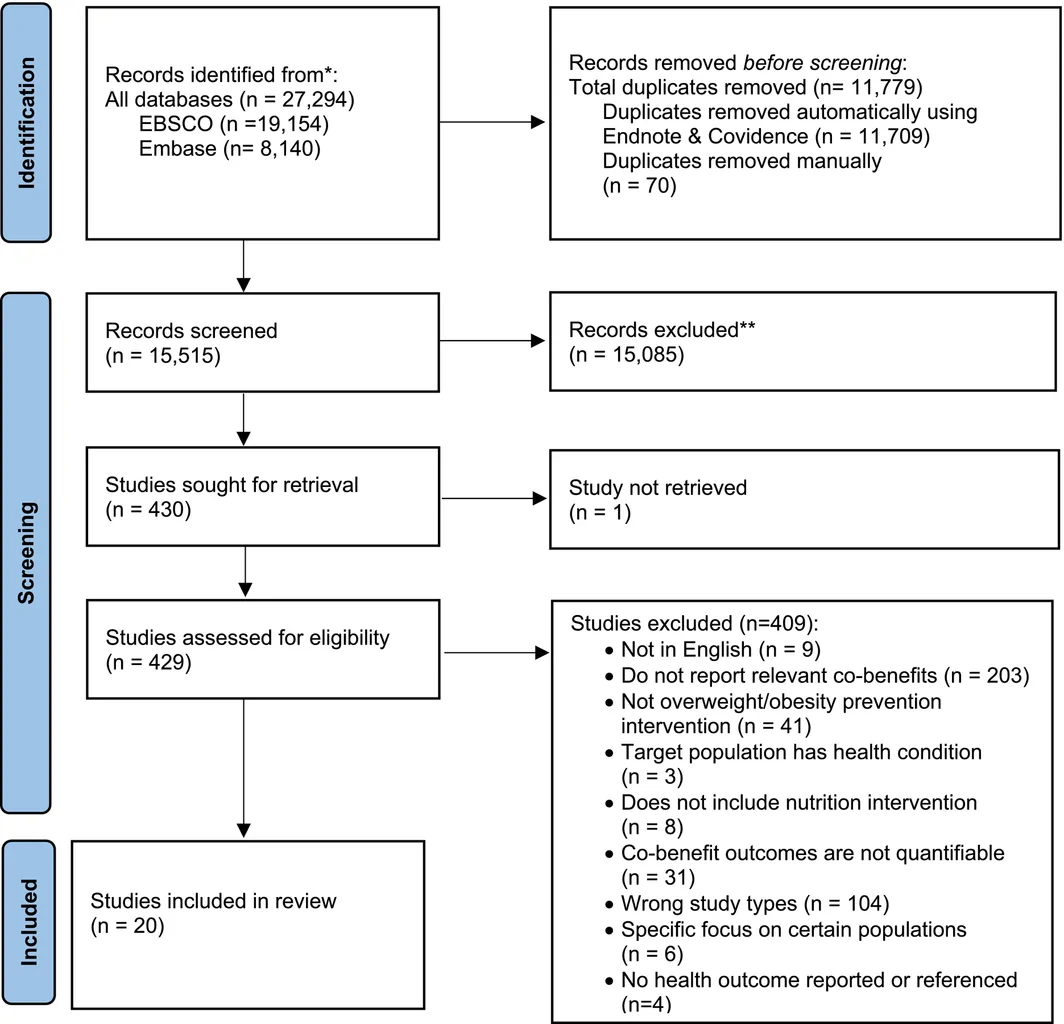
PRISMA flow diagram.

There were a range of study designs. There were four longitudinal retrospective studies [26, 28, 29, 36], four modeling studies [39, 40, 41, 42], one RCT [33], two cluster RCTs [23, 30], six pre‐post intervention studies [24, 25, 31, 34, 35], two quasi‐experiment studies [27, 32], and one cohort survey study [37].

The key aims for the nutrition interventions were to improve diet quality and/or overweight/obesity prevention. The interventions were set in schools (*n* = 9) [23, 24, 25, 26, 27, 28, 29, 30, 31], health center (*n* = 1) [35], communities (*n* = 4) [33, 34, 36, 37], and online (*n* = 1) [43]. One study [32] implemented an intervention across multiple settings (homes, schools, and supermarkets). The three modeling studies utilized national survey data to report results for the population as a whole [39, 40, 41].

Ten studies quantified educational co‐benefits [23, 24, 25, 26, 27, 28, 29, 30, 31, 32], eight studies reported social co‐benefits [23, 25, 33, 34, 35, 36, 43], and four studies reported environmental co‐benefits [37, 38, 39, 40, 41]. Table 1 below illustrates the type of co‐benefits reported in included studies. Eleven out of 20 (55%) studies reported positive impacts (i.e., statistically significant improvement) on co‐benefits [23, 24, 25, 26, 27, 33, 34, 38, 39, 40, 41]. Nine studies (45%) reported no significant improvement, or mixed results (i.e., significant improvement was observed in parts of the population, but not all) [28, 29, 30, 31, 32, 35, 36, 37, 43]. Further details on interventions and results are reported in the next sections.

**TABLE 1:** Summary of the co‐benefits of preventive nutrition interventions.

Author (year)	Population, setting, study design, type of intervention	Co‐benefits			Quality assessment
		Educational	Social	Environmental	
Melnyk 2013 [23]	Population: Adolescents; setting: Schools (*n* = 779); country: USA; design: Cluster RCT; multicomponent school‐based intervention	Positive impact	Positive impact		100% (MMAT)
Saxe‐Custack 2021 [24]	Population: Children (*n* = 186) mean age 10.55 ± 1.83 years; setting: Farmers' market; country: USA; design: Pre‐post study; cooking program	Positive impact	Positive impact		100% (MMAT)
Schultz 2022 [25]	Population: Students (*n* = 2000); setting: Schools and kindergarten; country: USA; design: Pre–post study; school‐based gardening program	Positive impact	Positive impact		71% (MMAT)
Destaw 2022 [26]	Population: Schoolchildren aged 5–19 years old (*n* = 4500); setting: School; country: Ethiopia; design: Longitudinal study; school feeding program	Positive impact			100% (MMAT)
Metwally 2020 [27]	Population: Students in the fifth grade (*n* = 1789); setting: School; country: Egypt; design: A quasi‐experimental study (post‐test with control group); school feeding program	Positive impact			57% (MMAT)
Bullock 2022 [28]	Population: Students, age group not specified (*n* = 150 schools); setting: Elementary, middle, and high schools; country: USA; design: Longitudinal study; school feeding program	No impact			100% (MMAT)
Corcoran 2016 [29]	Population: Students, age group not specified (*n* = 1088); setting: Elementary and middle schools; country: USA; design: Longitudinal study; school feeding program	No impact			85% (MMAT)
Hernandez 2014 [30]	Population: Students from sixth to ninth grade, aged 11–14 years old (*n* = 4603); setting: School; country: USA; design: Cluster RCT; multicomponent school‐based intervention	No impact			85% (MMAT)
Melnyk 2014 [31]	Population: First‐year college students, aged 18 to 21 years old (*n* = 49); setting: College; country: USA; design: Pre–post study; multicomponent school‐based intervention	No impact			100% (MMAT)
Treu 2017 [32]	Population: Students in third grade (*n* = 1487); setting: Elementary schools, homes, and a supermarket; country: USA; design: Quasi‐experimental with three arms; multicomponent school‐based intervention	Mixed results			57% (MMAT)
Gesell 2012 [33]	Population: Latino families with preschool aged children (*n* = 305); setting: Public recreation center; country: USA; design: RCT; community‐based multicomponent intervention.		Positive impact		85% (MMAT)
Raine 2013 [34]	Population: Adults (*n* = 14,517); setting: “Healthy Alberta Communities” HAC communities; country: Canada; design: pre–post study; community‐based multicomponent intervention		Positive impact		85% (MMAT)
Izumi 2020 [35]	Population: English‐ and Spanish‐speaking patients of Mid County Health Center (*n* = 48); setting: Mid County Health Center; country: USA; design: pre–post study; produce share from government funded farming program		No impact		85% (MMAT)
Korn 2018 [36]	Population: Stakeholder group members (*n* = 153); setting: Not applicable; country: Australia; design: Longitudinal survey; school/community‐based multi component intervention		No impact		71% (MMAT)
Ranjit 2020 [37]	Population: Parent–child dyads (*n* = 313); setting: community; country: USA; design: A 4‐year evaluation study and cohort‐control study; community‐based multi component intervention		Mixed results		100% (MMAT)
Colombo 2024 [42]	Population: adults aged 18 years (*n* = 24,293); setting: Not applicable; country: UK; design: tax scenarios on food groups modeling			Positive impact	100% (MMAT)
Monsivais 2015 [38]	Population: adults aged 39–79 years (*n* = 24,293); setting: Not applicable; country: UK; design: Cross‐sectional study with diet scenarios modeling			Positive impact	85% (MMAT)
Wang 2021 [39]	Population: Adults aged 18–75 (*n* = 38,070); setting: Not applicable; country: China; design: scenarios analyses modeling study			Positive impact	100% (MMAT)
Caillavet 2016 [40]	Population: French households between 1998–2010 (*n* = 48 cohorts); setting: Not applicable; country: France; Design: tax scenarios on food groups modeling			Positive impact	100% (MMAT)
Castiglione 2019 [41]	Population: UK population; setting: Not applicable; country: UK; design: Pre–post study, community‐based fruit and vegetable intake intervention			Positive impact	100% (MMAT)

*Note:* MMAT: Mixed Methods Appraisal Tool.

### 3.2 Educational co‐Benefits

Ten out of 20 included studies reported educational co‐benefits. Five studies reported favorable educational impacts, four studies reported no significant impact, and one study reported mixed results.

#### 3.2.1 Type of Educational Co‐Benefits and Outcome Measurement

Of the 10 studies that reported educational co‐benefits, six reported academic performance [23, 26, 27, 29, 30, 32], and eight studies reported academic behavior [24, 25, 26, 28, 29, 30, 31, 32], with four studies reporting both outcomes. Among the six studies evaluating academic performance, 66% reported a positive impact across all measures. Conversely, 33% indicated either non‐significant changes or inconsistent positive effects among sub‐groups. One study yielded mixed results, demonstrating positive impacts on certain outcomes (reading tests) but significantly less favorable outcomes (mathematics tests) for the intervention group in other areas [32]. Of the eight studies assessing academic behavior, 50% reported significant improvements, whereas the remaining 50% indicated non‐significant improvements. The school feeding programs appeared to enhance academic performance in economically disadvantaged populations, including those in Ethiopia [26], Egypt [27], and certain regions of the USA [28, 29]. Table S2 reports the key characteristics of studies reporting impacts on educational co‐benefits.

The outcome measurement instruments used in studies examining educational co‐benefits varied. For academic behavior, the majority of studies used administrative data on attendance rates [26, 28, 29, 30, 31], disciplinary action [30], or college retention [31]. For academic performance, three studies reported use of administrative data for either math and reading tests [30], or average semester scores [26, 29]. Five studies used published scales or tools to measure academic performance [23, 24, 25, 27, 32].

#### 3.2.2 Type of Interventions

Four of the 10 studies were school feeding programs [26, 27, 28, 29], four studies were multicomponent interventions which usually consisted of physical activity, nutrition, classroom behavior, education, and emotional and/or behavioral coping strategies [23, 26, 30, 31, 32], one was a gardening program [25], and one study was a cooking program for students [24]. Fifty percent of the school feeding programs were from developing countries, which also reported positive significant impacts on both performance and behavior, while the other 50% were conducted in the United States and reported non‐significant impacts.

Among four multicomponent interventions, all conducted in the United States, one study reported positive impacts on academic performance [23], another two studies [30, 31] reported non‐significant improvement.

Both the gardening school program and the school cooking program studies reported improved academic behavior [24, 25]. However, only Saxe‐Custack et al. [24] reported a statistically significant improvement in academic self‐efficacy [24]. While Schultz et al. [25] reported improved school behavior in terms of academic self‐efficacy for the gardening‐education program, statistical significance was not reported [25].

### 3.3 Social Co‐Benefits

Eight studies reported social co‐benefits [23, 24, 25, 33, 34, 35, 36, 37]. Overall, five studies (63%) reported significant improvement in social co‐benefits [23, 24, 25, 33, 34], while another two studies [35, 36] reported non‐significant improvement. One study observed a decline in perception of safety in the community [37].

#### 3.3.1 Type of Social Co‐Benefits and Outcomes Measurement

Studies have utilized diverse methodologies to measure social co‐benefits of nutrition interventions: social connection as in social ties/socializing/social networking (three studies [33, 36]), sense of belonging [34], social functioning/self‐efficacy, social and emotional capacities (two studies [23, 34]), and perception of neighborhood safety [37].

Surveys were the measurement instrument used in all studies examining social co‐benefits. Four out of eight studies used validated instruments, all of which used different measurements of outcomes: positive identity [25], interacting with other children [24] and socializing and taking part in social activities [35]. In the four studies that did not use validated instruments, two undertook social networking surveys [33, 36], one study measured sense of belonging [34] and another study reported on socio‐environmental perceptions [37].

#### 3.3.2 Type of Intervention

Four studies that reported social co‐benefits were multicomponent interventions, that is, employed nutrition component and PA or other components such as screen time or built environment [23, 33, 34, 36], one study was a school gardening program [25], one study assessed a subsidized grant program for improving food security [35], and one study assessed a school cooking program [24]. Characteristics of studies reporting social co‐benefits are reported in Table S3. Raine et al. (2013) [34] reported that sense of belonging significantly improved in the multicomponent intervention group compared to baseline and the control group. Melnyk et al. (2013) [23] reported no significant change between intervention and control group for anger and destructive behavior from a multicomponent intervention. Another multicomponent intervention reported participants had a significantly poorer perception of neighborhood safety [37]. In a school gardening program, one study reported higher social emotional capacities (positive identity, contribution and social skills) from baseline to follow‐up but statistical significance was not reported [25].

In terms of target population, one study targeted parent–child dyads, that is, pairs consisting of a parent and their child, emphasizing the interaction and influence between the two individuals in shaping behaviors or outcomes [33]. Another study recruited stakeholder organizations/institutions (kindergarten associations, local department of, department of human services, etc.) [36] within a community, two studies targeted school students [23, 25], another study focused on people aged 18 years and over living in non‐institutionalized settings, one study targeted clients from a medical clinic [35], and another study included participants 60 years and older [23, 25, 33, 34, 35, 36].

### 3.4 Environmental co‐Benefits

Five studies reported on environmental outcomes of changing population diet, all of which used modeling approaches to estimate emissions [38, 39, 40, 41, 42]. The environmental co‐benefits reported included GHGE like carbon dioxide (CO_2_) (*n* = 5) [38, 39, 40, 41, 42], sulfur dioxide (SO_2_) (*n* = 2) [40, 42], phosphate (PO_4_) (*n* = 1) [42], and nitrogen dioxide (N) (*n* = 1) [40]. Colombo et al. (2024) [42] also reported changes in land use, water use, and water scarcity. Characteristics of studies reporting environmental co‐benefits are reported in Table S4.

All five studies consistently reported significant reductions in GHGEs [38, 39, 40, 41]. Caillavet et al. (2016) [40] reported more reduction in CO_2_ and SO_2_ for taxation of all animal‐based food products compared to studies that reported more environmentally friendly diets [38, 39, 41]. Colombo et al. (2024) [42] similarly found that substituting high‐sugar and high‐salt foods with lower‐content alternatives produced beneficial effects on emissions, land use, and water use.

Each study used a different source for GHGEs associated with diets. Wang et al. (2021) [39] used the Intergovernmental Panel on Climate Change and calculated GHGEs by multiplying emission factors by activity data, whereas Caillavet et al. (2016) [40] used a Life Cycle Analysis to estimate products or activities in terms of environmental impact. Monsivais et al. (2015) [38] estimated potential GHGEs for each of 289 food codes by applying 100‐year global warming potential weightings to CO_2_, methane, and N, using life‐cycle emission factors. Meanwhile Castiglione et al. (2019) [41] calculated GHGEs using supply‐chain life‐cycle emission factors incorporating emissions from production to retail distribution. Colombo et al. (2024) used published meta‐analysis of food product Life Cycle Assessments to estimate environmental impacts associated with specific food products [42].

### 3.5 Quality Assessment of All Included Studies

No studies were excluded based on the quality assessment. Of the 20 studies assessed using the MMAT tool, the majority fulfilled all criteria (*n* = 9) [23, 24, 26, 28, 31, 37] or most criteria (average score = 6/7, *n* = 6) [29, 30, 33, 34, 35, 38]. Only two studies scored 5 out of 7 favorable responses [25, 36] and two other studies scored 4 “yes” out of 7 favorable responses [27, 32]. For non‐randomized studies, reporting was limited and only assessed the following questions: “Are there complete data?” and “Are the confounders accounted for in the design and analysis?.” For randomized controlled trial studies, “Are outcome assessors blinded to the intervention provided?” reported the lowest responses.

## 4 Discussion

In summary, out of the 20 studies included in this review, educational co‐benefits were the most commonly reported outcome (10 studies [23, 24, 25, 26, 27, 28, 29, 30, 31, 32]) followed by social co‐benefits (eight studies [23, 24, 25, 33, 34, 35, 36, 37]) and environmental co‐benefits (five studies [38, 39, 40, 41]). All studies reporting environmental co‐benefits consistently reported statistically significant positive impacts of nutrition interventions on environmental outcomes, while intervention impacts on educational and social co‐benefits had mixed results. Interestingly, three school‐based interventions [23, 24, 25] that investigated co‐benefits on both educational and social benefits reported positive impacts on both outcomes.

Educational co‐benefits primarily relate to improvements in academic performance and attendance. Integrating nutrition programs within schools and early childhood settings can support learning outcomes while reinforcing healthy behaviors [26, 29]. These educational impacts can be assessed efficiently through linked administrative datasets within the education sector, enabling broader, low‐burden measurement of educational co‐benefits. Policies that embed nutrition education into curricula and strengthen food environments in educational institutions may therefore advance both health and educational outcomes [12].

Social co‐benefits were commonly observed in community‐based interventions and were highly diverse, encompassing outcomes such as social ties, peer interaction, community engagement, and reductions in feelings of loneliness. This pattern highlights the potential for nutrition initiatives embedded in community contexts to enhance social cohesion and collective well‐being beyond their primary health objectives. In a recent study, Petresin et al. (2025) [44] reported that participants in a food‐access intervention also valued the social interaction and sense of belonging that resulted from the intervention. Similarly, a review by Spezia et al. (2024) [45] highlighted “sense of community” as both an outcome and a driver of engagement for health promotion in community settings but noted the evidence remains limited. This synthesis highlighted conceptual and methodological challenges in measuring social co‐benefits. Similarly, variability in capturing social co‐benefits has been noted in the broader public‐health research, where challenges in definitions and outcome measures hinder cross‐study comparison. As reported in previous studies by Brown et al. (2022) [46], this lack of standardization constrains synthesis and limits identification of the most effective intervention components [45].

Studies assessing environmental outcomes in this review consistently reported significant reductions in GHGEs, reinforcing the potential of nutrition interventions, particularly fiscal and upstream food‐environment policies, to deliver dual health and planetary benefits [40, 42]. These co‐benefits (economic, social, and environmental sustainability) are now being incorporated into the development of the revised Australian National Dietary Guidelines [47, 48]. However, heterogeneity in modeling methods and data sources, including the use of diverse life‐cycle assessment frameworks, inherently contributes to variability in the magnitude of reported effects.

The potential for nutrition interventions to have broader impacts and therefore the consideration of these impacts into intervention design and measurement are increasingly recognized. Ward et al. (2025) [49] reported measurable social and emotional co‐benefits, such as improved self‐management and social skills, when climate and community dimensions were integrated into obesity‐prevention intervention design. Recent studies have also begun to develop frameworks to integrate social and environmental indicators into nutrition evaluation. Petruzzelli et al. (2025) [50], for example, proposed a comprehensive monitoring tool combining nutritional, environmental, and social sustainability metrics, addressing one of the major methodological gaps highlighted in this review. Future research should develop Core Outcome Sets as guidance on the most appropriate measurement tools to measure the co‐benefits of preventive health initiatives.

The small number of eligible studies in this review restricts the robustness of conclusions. The limited yield likely reflects the novelty of the co‐benefits concept. This limited evidence base prevented meta‐analysis and restricted generalizability. Furthermore, this review focused exclusively on three non‐health co‐benefit domains: educational, social, and environmental, and did not include other co‐benefit domains, such as economic (productivity and future healthcare cost saving) or food security and equity [12, 51]. As such, the findings may not capture the full spectrum of potential societal gains from nutrition interventions. Expanding future reviews to include additional domains would provide a more comprehensive understanding of the broader impacts of nutrition strategies.

This review focused specifically on nutrition interventions and policies aimed at preventing obesity and chronic diseases that reported changes in physical or clinical health as primary outcomes. As a result, many qualitative studies and process evaluations were excluded because they did not assess measurable health effects. Although qualitative studies were outside our scope, they can offer valuable insights into implementation processes, social dynamics, and contextual mechanisms that contribute to the identification of co‐benefits, particularly for co‐benefits that are not yet well conceptualized in the literature or for which validated measurement tools are still lacking [52].

Despite the limitations of this study, this review can be viewed as an essential step into planning future studies that identify, measure and value co‐benefits, prioritization of types of co‐benefits to value and standardization of outcomes measured. By valuing all impacts of interventions, we will enable better prioritization of interventions that optimize overall societal outcomes and encourage collaboration between different sectors across government departments to implement effective policies. Our findings highlight the need for more innovative methods to quantify co‐benefits and improved access to administrative datasets (e.g., for educational co‐benefits) to facilitate the evaluation of a broader range of intervention impacts.

## 5 Conclusion

This review synthesizes emerging evidence on the broad societal value of nutrition interventions, finding consistently positive impacts on environmental sustainability. However, positive educational and social outcomes were not found across the studies. Quantifying co‐benefits can support more holistic investment decisions and encourage collaboration between health, education, and environmental sectors. Future studies should build on methodological innovations, advancing toward integrated evaluation frameworks that capture the full spectrum of benefits arising from nutrition and food environment actions using consistent and validated tools.

## Author Contributions

J.A., V.B., and P.N. conceptualized the study. S.K.B.M.A. conducted the bibliographic search and, along with P.N., N.W., M.A.S., and M.S., carried out paper screening, quality assessment, and data extraction. P.N. and S.M.A. performed the synthesis, interpreted the results, and drafted the manuscript. L.V., A.M., and B.Z.‐D. contributed to study design, provided methodological input, and assisted with manuscript editing. All authors reviewed and approved the final version of the manuscript.

## Funding

The project was funded by The Australian Prevention Partnership Centre. P.N. is supported by a Deakin University Postgraduate Fellowship. J.A. is supported by a Deakin University Postgraduate Fellowship and an NHMRC Investigator Grant (APP 2033338). M.S. is supported by a Deakin University Postdoctoral Research Fellowship. V.B. is supported by NHMRC grants GNT2006999 and GNT2002234. Funding organizations had no role in the systematic review process.

## Conflicts of Interest

The authors declare no conflicts of interest.

## Supporting information




**Table S1:** Summary of study characteristics.
**Table S2:** Summary of studies reporting educational co‐benefits.
**Table S3:** Summary of studies reporting social co‐benefits.
**Table S4:** Summary of studies reporting environmental co‐benefits.

## Data Availability

The data that support the findings of this study are available in the Supporting Information of this article.

## References

[1] E. Nury , J. Stadelmaier , J. Morze , et al., “Effects of Nutritional Intervention Strategies in the Primary Prevention of Overweight and Obesity in School Settings: Systematic Review and Network Meta‐Analysis,” BMJ Medicine 1 (2022): e000346, 10.1136/bmjmed-2022-000346. PMC9951385 36936562

[2] World Health Organization World Health Statistics 2024 2024.

[3] Institute for Health Metrics and Evaluation (IHME) Global Burden of Disease 2021: Findings From the GBD 2021 Study 2024 https://www.healthdata.org/research‐analysis/library/global‐burden‐disease‐2021‐findings‐gbd‐2021‐study.

[4] GBD 2019 Diseases and Injuries Collaborators , “Global Burden of 369 Diseases and Injuries in 204 Countries and Territories, 1990–2019: A Systematic Analysis for the Global Burden of Disease Study 2019,” Lancet 396, no. 10258 (2020): 1204–1222, 10.1016/s0140-6736(20)30925-9. PMC7567026 33069326

[5] Institute for Health Metrics and Evaluation (IHME) , Global Burden of Disease 2023: Findings From the GBD 2023 study (IHME, 2025), https://www.healthdata.org/research‐analysis/library/global‐burden‐disease‐2023‐findings‐gbd‐2023‐study.

[6] World Health Organization , Fiscal policies to promote healthy diets: WHO guideline (World Health Organization, 2024), 120. https://www.who.int/publications/i/item/9789240091016. 38976625

[7] World Health Organization , Global Action Plan for the Prevention and Control of Noncommunicable Diseases (NCDs) 2013–2020 (Extended to 2030) (World Health Organization, 2013), 55.

[8] I. P. Nadal , C. Angkurawaranon , A. Singh , et al., “Effectiveness of Behaviour Change Techniques in Lifestyle Interventions for Non‐Communicable Diseases: An Umbrella Review,” BMC Public Health 24, no. 1 (2024): 3082, 10.1186/s12889-024-20612-8. PMC11545567 39511525

[9] H. Koorts , J. Ma , C. T. V. Swain , H. Rutter , J. Salmon , and K. A. Bolton , “Systems Approaches to Scaling up: A Systematic Review and Narrative Synthesis of Evidence for Physical Activity and Other Behavioural Non‐Communicable Disease Risk Factors,” International Journal of Behavioral Nutrition and Physical Activity 21, no. 1 (2024): 32, 10.1186/s12966-024-01579-6. PMC10958859 38515118

[10] The Australian Prevention Partnership Centre . What are the co‐benefits of prevention? (Sax Institute, 2023). Accessed 16/10/2023, https://preventioncentre.org.au/about‐prevention/what‐are‐the‐co‐benefits‐of‐prevention/.

[11] The Australian Prevention Partnership Centre Multisectoral collaboration and its impact on health and wellbeing (Sax Institute, 2023).

[12] Howse E Crosland P Rychetnik L Wilson A The Value of Prevention: An Evidence Check Rapid Review The Australian Prevention Partnership Centre 2021.

[13] Q. Rachmah , T. Martiana , n. Mulyono , et al., “The Effectiveness of Nutrition and Health Intervention in Workplace Setting: A Systematic Review,” Journal of Public Health Research 11, no. 1 (2022): jphr.2021.2312, 10.4081/jphr.2021.2312. PMC8859724 34781627

[14] C. Burgaz , V. Gorasso , W. M. J. Achten , et al., “The Effe Nutrition, Nutrition‐Related Inequalities and Environmental Sustainability: A Scoping Review,” Food Security 15, no. 5 (2023): 1313–1344, 10.1007/s12571-023-01385-1.

[15] B. A. Swinburn , V. I. Kraak , S. Allender , et al., “The Global Syndemic of Obesity, Undernutrition, and Climate Change: *The Lancet* Commission Report,” Lancet 393, no. 10173 (2019): 791–846, 10.1016/S0140-6736(18)32822-8. 30700377

[16] M. J. Page , J. E. McKenzie , P. M. Bossuyt , et al., “The PRISMA 2020 Statement: An Updated Guideline for Reporting Systematic Reviews,” BMJ 372 (2021): n71, 10.1136/bmj.n71. PMC8005924 33782057

[17] World Health Organization . Healthy diet (WHO, 2020), https://www.who.int/news‐room/fact‐sheets/detail/healthy‐diet.

[18] The EndNote Team , EndNote 20 (Clarivate, 2013).

[19] The Covidence Team . Covidence systematic review software, Veritas Health Innovation, Melbourne, Australia, https://www.covidence.org/.

[20] M. Campbell , J. E. McKenzie , A. Sowden , et al., “Synthesis Without Meta‐Analysis (SWiM) in Systematic Reviews: Reporting Guideline,” BMJ 368 (2020): l6890, 10.1136/bmj.l6890. PMC7190266 31948937

[21] Q. N. Hong , S. Fàbregues , G. Bartlett , et al., “The Mixed Methods Appraisal Tool (MMAT) Version 2018 for Information Professionals and Researchers,” Education for Information 34, no. 4 (2018): 285–291.

[22] R. Pace , P. Pluye , G. Bartlett , et al., “Testing the Reliability and Efficiency of the Pilot Mixed Methods Appraisal Tool (MMAT) for Systematic Mixed Studies Review,” International Journal of Nursing Studies 49, no. 1 (2012): 47–53, 10.1016/j.ijnurstu.2011.07.002. 21835406

[23] B. M. Melnyk , D. Jacobson , S. Kelly , et al., “Promoting Healthy Lifestyles in High School Adolescents: A Randomized Controlled Trial,” American Journal of Preventive Medicine 45, no. 4 (2013): 407–415, 10.1016/j.amepre.2013.05.013. PMC4285557 24050416

[24] A. Saxe‐Custack , J. LaChance , M. Hanna‐Attisha , and C. Dawson , “Flint Kids Cook: Positive Influence of a Farmers' Market Cooking and Nutrition Programme on Health‐Related Quality of Life of US Children in a Low‐Income, Urban Community,” Public Health Nutrition 24, no. 6 (2021): 1492–1500, 10.1017/S136898002000395X. PMC10195638 33028450

[25] C. Schultz and A. E. Rosen , “School Gardens' Impact on Students' Health Outcomes in Low‐Income Midwest Schools,” Journal of School Nursing: The Official Publication of the National Association of School Nurses 38, no. 5 (2022): 486–493, 10.1177/10598405221080970. 35213238

[26] Z. Destaw , E. Wencheko , S. Kidane , et al., “Impact of School Meals on Educational Outcomes in Addis Ababa, Ethiopia,” Public Health Nutrition 25, no. 9 (2022): 2614–2624, 10.1017/S1368980022000799. PMC9991771 35343425

[27] A. M. Metwally , M. M. El‐Sonbaty , L. A. El Etreby , et al., “Impact of National Egyptian School Feeding Program on Growth, Development, and School Achievement of School Children,” World Journal of Pediatrics: WJP 16, no. 4 (2020): 393–400, 10.1007/s12519-020-00342-8. 32056148

[28] S. L. Bullock , S. Dawson‐McClure , K. P. Truesdale , D. S. Ward , A. E. Aiello , and A. S. Ammerman , “Associations Between a Universal Free Breakfast Policy and School Breakfast Program Participation, School Attendance, and Weight Status: A District‐Wide Analysis,” International Journal of Environmental Research and Public Health 19, no. 7 (2022): 3749, 10.3390/ijerph19073749. PMC8998064 35409433

[29] S. P. Corcoran , B. Elbel , and A. E. Schwartz , “The Effect of Breakfast in the Classroom on Obesity and Academic Performance: Evidence From New York City,” Journal of Policy Analysis and Management: [the Journal of the Association for Public Policy Analysis and Management] 35, no. 3 (2016): 509–532, 10.1002/pam.21909. PMC4977577 27314139

[30] A. E. Hernandez , M. D. Marcus , K. Hirst , M. S. Faith , L. Goldberg , and R. P. Treviño , “Impact of Implementation and Conduct of the HEALTHY Primary Prevention Trial on Student Performance,” American Journal of Health Promotion: AJHP 29, no. 1 (2014): 55–58, 10.4278/ajhp.130131-ARB-53. PMC4751880 24200256

[31] B. Melnyk , S. Kelly , D. Jacobson , K. Arcoleo , and G. Shaibi , “Improving Physical Activity, Mental Health Outcomes, and Academic Retention in College Students With Freshman 5 to Thrive: COPE/Healthy Lifestyles,” Journal of the American Association of Nurse Practitioners 26, no. 6 (2014): 314–322, 10.1002/2327-6924.12037. 24170429

[32] J. A. Treu , K. Doughty , J. S. Reynolds , V. Y. Njike , and D. L. Katz , “Advancing School and Community Engagement Now for Disease Prevention (ASCEND): A Quasi‐Experimental Trial of School‐Based Interventions to Prevent Childhood Obesity,” American Journal of Health Promotion 31, no. 2 (2017): 143–152, 10.4278/ajhp.140820-QUAN-413. 26559709

[33] S. B. Gesell , K. D. Bess , and S. L. Barkin , “Understanding the Social Networks That Form Within the Context of an Obesity Prevention Intervention,” Journal of Obesity 2012 (2012): 749832, 10.1155/2012/749832. PMC3359823 22655175

[34] K. Raine , R. Plotnikoff , D. Schopflocher , et al., “Healthy Alberta Communities Impact of a Three‐Year Community‐Based Obesity and Chronic Disease Prevention Intervention,” Obesity Research and Clinical Practice 7 (2013): e106, 10.1016/j.orcp.2013.12.690. 24016521

[35] B. T. Izumi , A. Martin , T. Garvin , et al., “CSA Partnerships for Health: Outcome Evaluation Results From a Subsidized Community‐Supported Agriculture Program to Connect Safety‐Net Clinic Patients With Farms to Improve Dietary Behaviors, Food Security, and Overall Health,” Translational Behavioral Medicine 10, no. 6 (2020): 1277–1285, 10.1093/tbm/ibaa041. 33421087

[36] A. R. Korn , E. Hennessy , R. A. Hammond , et al., “Development and Testing of a Novel Survey to Assess Stakeholder‐Driven Community Diffusion of Childhood Obesity Prevention Efforts,” BMC Public Health 18, no. 1 (2018): 681, 10.1186/s12889-018-5588-1. PMC5984309 29855295

[37] N. Ranjit , A. Nielsen , N. Akhavan , et al., “Outcomes of a Community‐Wide Health Intervention in a Low‐Income, Primarily Hispanic Community: The Go! Austin/Vamos! Austin (Gava) Initiative,” Health Promotion Practice 23, no. 1 (2020): 185–194, 10.1177/1524839920961365. 33034208

[38] P. Monsivais , P. Scarborough , T. Lloyd , et al., “Greater Accordance With the Dietary Approaches to Stop Hypertension Dietary Pattern Is Associated With Lower Diet‐Related Greenhouse Gas Production but Higher Dietary Costs in the United Kingdom,” American Journal of Clinical Nutrition 102, no. 1 (2015): 138–145, 10.3945/ajcn.114.090639. PMC4480663 25926505

[39] L. Wang , S. Cui , Y. Hu , et al., “The Co‐benefits for Food Carbon Footprint and Overweight and Obesity From Dietary Adjustments in China,” Journal of Cleaner Production 289 (2021): 125675, 10.1016/j.jclepro.2020.125675, https://www.sciencedirect.com/science/article/abs/pii/S0959652620357218.

[40] F. Caillavet , A. Fadhuile , and V. Nichèle , “Taxing Animal‐Based Foods for Sustainability: Environmental, Nutritional and Social Perspectives in France,” European Review of Agricultural Economics 43, no. 4 (2016): 537–560, 10.1093/erae/jbv041, https://erae.oxfordjournals.org/content/43/4/537.abstract.

[41] C. Castiglione and M. Mazzocchi , “Ten Years of Five‐A‐Day Policy in the UK: Nutritional Outcomes and Environmental Effects,” Ecological Economics 157 (2019): 185–194, 10.1016/j.ecolecon.2018.11.016.

[42] P. E. Colombo , R. Green , S. Nájera Espinosa , G. Scarpa , R. Saha , and P. Scheelbeek , “Modelling Impacts of a Salt and Sugar Tax on Hypothetical Intra‐category Food Substitutions, BMI and Environmental Footprints in the UK Population,” European Journal of Nutrition 63, no. 7 (2024): 2643–2653. 10.1007/s00394-024-03452-5 PMC11490524 38935140

[43] G. McKeon , A. Tiedemann , C. Sherrington , et al., “Feasibility of an Online, Mental Health‐Informed Lifestyle Program for People Aged 60+ Years During the COVID‐19 Pandemic,” Health Promotion Journal of Australia: Official Journal of Australian Association of Health Promotion Professionals 33, no. 3 (2022): 545–552, 10.1002/hpja.538. 34496102

[44] T. Petresin , M. Any , N. Shujauddin , A. Annis , J. Haines , and f.u.n. Community A , “Food Uniting Neighbours (f.u.n.): A Pilot Study Exploring the Feasibility, Acceptability, and Preliminary Impact of Co‐Designed Interventions to Improve Food Access,” BMC Public Health 25, no. 1 (2025): 2086, 10.1186/s12889-025-23356-1. PMC12139161 40468223

[45] N. Spezia , S. De Rosis , and S. Nuti , “Sense of Community in the Context of Disease Prevention and Health Promotion: A Scoping Review of the Literature,” BMC Public Health 24, no. 1 (2024): 3090, 10.1186/s12889-024-20515-8. PMC11546476 39516795

[46] V. Brown , M. Moodie , M. Sultana , et al., “A Scoping Review of Outcomes Commonly Reported in Obesity Prevention Interventions Aiming to Improve Obesity‐Related Health Behaviors in Children to Age 5 Years,” Obesity Reviews 23, no. 5 (2022): e13427, 10.1111/obr.13427. 35122457

[47] C. Dooren , B. Loken , T. Lang , et al., “The Planet on Our Plates: Approaches to Incorporate Environmental Sustainability Within Food‐Based Dietary Guidelines,” Frontiers in Nutrition 11 (2024): 1223814, 10.3389/fnut.2024.1223814. PMC11259098 39036493

[48] National Health and Medical Research Council . NHMRC Update on the Australian Dietary Guidelines (NHMRC, 2025). Accessed 28/11, 2025, https://www.nhmrc.gov.au/about‐us/news‐centre/nhmrc‐update‐australian‐dietary‐guidelines.

[49] N. Ward , K. Robinson , J. Jacobs , M. Nichols , M. Moodie , and V. Brown , “Including Climate Change in Community‐Based Obesity Prevention Interventions: A Qualitative Exploration of the Perspectives of Australian Funders,” BMC Public Health 25, no. 1 (2025): 1526, 10.1186/s12889-025-22599-2. PMC12020184 40275219

[50] M. Petruzzelli , S. Amadori , R. Ihle , M. Fridel , and M. Vittuari , “Monitoring Nutritional, Environmental and Social Sustainability in School Food Settings: A Three‐Dimensional Score‐Based Assessment Tool,” Journal of Cleaner Production 519 (2025): 146015, 10.1016/j.jclepro.2025.146015.

[51] J. Hammaker , D. Anda , T. Kozakiewicz , et al., “Systematic Review on Fiscal Policy Interventions in Nutrition. Systematic Review,” Frontiers in Nutrition 9 (2022): 967494, 10.3389/fnut.2022.967494. PMC9756132 36532551

[52] J. White , K. Thompson , J. Talwar , and D. N. Durrheim , “The Importance of Qualitative Social Science Research for Informed Public Health Policy at Local and National Levels: Insights From a Local Health District in New South Wales, Australia. Perspective,” Frontiers in Public Health 13 (2025): 1575188, 10.3389/fpubh.2025.1575188. PMC12098541 40416664

