# Supplementary material for: Co‐Benefits of Nutrition Interventions: A Systematic Review
Source: Obes Rev. 2026 May 11;27(12):e70159. doi: 10.1111/obr.70159 (PMC13749371; doi:10.1111/obr.70159)
Supplement: Supplementary file 1 [file OBR-27-e70159-s001.pdf]

Co-benefits of nutrition interventions: a systematic review

Authors

Phuong Nguyen [1,2]

Siti Khadijah binti Mohamad Asfia [3]

Vicki Brown [1,2]

Nicole Ward [1,2]

Moosa AlSubhi [1,2]

Marufa Sultana [1,2]

Lennert Veerman [4]

Andrew Milat [5]

Belen Zapata-Diomedi [6]

Jaithri Ananthapavan [1,2]

Affiliations

[1] Deakin Health Economics, Institute for Health Transformation, School of Health and Social Development, Faculty of Health, Deakin University, Burwood, Victoria, 3125 Australia

[2] Global Centre for Preventive Health and Nutrition, Institute for Health Transformation, School of Health and Social Development, Faculty of Health, Deakin University, Burwood, Victoria, 3125 Australia

[3] Ministry of Women, Early Childhood and Community Wellbeing Development, Bangunan Baitulmakmur, 2, Tingkat 8, Petra Jaya, 93050 Kuching, Sarawak, Malaysia

[4] Public Health & Economics Modelling group, School of Medicine and Dentistry, Griffith University

[5] Prevention Research Collaboration, The University of Sydney

[6] Centre for Urban Research, RMIT University

Correspondence: Dr Phuong Nguyen; email: phuong.nguyen@deakin.edu.au

Address: Deakin Health Economics, Deakin University

221 Burwood Highway, Burwood, Victoria 3125

Appendix

Include changes between protocol and systematic review

- No meta-analysis was conducted due to heterogeneity of studies
- Quality assessment tools used in systematic review are MMAT tool

SocINDEX search strategy

#	Query	Limiters/Expanders	Last Run Via
S98	S69 AND S83 AND S97	Limiters - Date of Publication: 20120101-20221231<br>Search modes - Boolean/Phrase	Interface - EBSCOhost Research Databases<br>Search Screen - Advanced Search<br>Database - SocINDEX with Full Text
S97	S84 OR S85 OR S86 OR S87 OR S88 OR S89 OR S90 OR S91 OR S92 OR S93 OR S94 OR S95 OR S96	Search modes - Boolean/Phrase	Interface - EBSCOhost Research Databases<br>Search Screen - Advanced Search<br>Database - SocINDEX with Full Text
S96	TI "high BMI" OR AB "high BMI"	Search modes - Boolean/Phrase	Interface - EBSCOhost Research Databases<br>Search Screen - Advanced Search<br>Database - SocINDEX with Full Text
S95	TI "high body mass index" OR AB "high body mass index"	Search modes - Boolean/Phrase	Interface - EBSCOhost Research Databases<br>Search Screen - Advanced Search<br>Database - SocINDEX with Full Text
S94	TI overweight OR AB overweight	Search modes - Boolean/Phrase	Interface - EBSCOhost Research Databases<br>Search Screen - Advanced Search<br>Database - SocINDEX with Full Text
S93	TI obes* OR AB obes*	Search modes - Boolean/Phrase	Interface - EBSCOhost Research Databases<br>Search Screen - Advanced Search<br>Database - SocINDEX with Full Text
S92	TI food N5 sustainab* OR AB food N5 sustainab*	Search modes - Boolean/Phrase	Interface – EBSCOhost Research Databases<br>Search Screen - Advanced<br>Search Database - SocINDEX with Full Text
S91	TI food N5 supplies OR AB food N5 supplies	Search modes - Boolean/Phrase	Interface - EBSCOhost Research Databases<br>Search Screen - Advanced Search<br>Database - SocINDEX with Full Text
S90	TI food N5 supply OR AB food N5 supply	Search modes - Boolean/Phrase	Interface - EBSCOhost Research Databases<br>Search Screen - Advanced Search<br>Database - SocINDEX with Full Text
S89	TI food N5 afford* OR AB food N5 afford*	Search modes - Boolean/Phrase	Interface - EBSCOhost Research Databases<br>Search Screen - Advanced Search<br>Database - SocINDEX with Full Text
S88	TI nutritional OR AB nutritional	Search modes - Boolean/Phrase	Interface - EBSCOhost Research Databases<br>Search Screen - Advanced Search<br>Database - SocINDEX with Full Text
S87	TI nutrition OR AB nutrition	Search modes - Boolean/Phrase	Interface - EBSCOhost Research Databases<br>Search Screen - Advanced Search<br>Database - SocINDEX with Full Text
S86	TI dietary OR AB dietary	Search modes - Boolean/Phrase	Interface - EBSCOhost Research Databases<br>Search Screen - Advanced Search<br>Database - SocINDEX with Full Text
S85	TI diets OR AB diets	Search modes - Boolean/Phrase	Interface - EBSCOhost Research Databases<br>Search Screen - Advanced Search<br>Database - SocINDEX with Full Text
S83	S70 OR S71 OR S72 OR S73 OR S74 OR S75 OR S76 OR S77 OR S78 OR S79 OR S80 OR S81 OR S82	Search modes - Boolean/Phrase	Interface - EBSCOhost Research Databases<br>Search Screen - Advanced Search<br>Database - SocINDEX with Full Text

#	Query	Limiters/Expanders	Last Run Via
S84	TI diet OR AB diet	Search modes - Boolean/Phrase	Interface - EBSCOhost Research Databases Search Screen - Advanced Search Database - SocINDEX with Full Text
S82	TI labelling OR AB labelling	Search modes - Boolean/Phrase	Interface - EBSCOhost Research Databases Search Screen - Advanced Search Database - SocINDEX with Full Text
S81	TI promot* OR AB promot*	Search modes - Boolean/Phrase	Interface - EBSCOhost Research Databases Search Screen - Advanced Search Database - SocINDEX with Full Text
S80	TI education OR AB education	Search modes - Boolean/Phrase	Interface - EBSCOhost Research Databases Search Screen - Advanced Search Database - SocINDEX with Full Text
S79	TI campaign* OR AB campaign*	Search modes - Boolean/Phrase	Interface - EBSCOhost Research Databases Search Screen - Advanced Search Database - SocINDEX with Full Text
S78	TI policies OR AB policies	Search modes - Boolean/Phrase	Interface - EBSCOhost Research Databases Search Screen – Advanced Search Database - SocINDEX with Full Text
S77	TI policy OR AB policy	Search modes - Boolean/Phrase	Interface - EBSCOhost Research Databases Search Screen - Advanced Search Database - SocINDEX with Full Text
S76	TI legislation OR AB legislation	Search modes - Boolean/Phrase	Interface - EBSCOhost Research Databases Search Screen - Advanced Search Database - SocINDEX with Full Text
S75	TI taxes OR AB taxes	Search modes - Boolean/Phrase	Interface - EBSCOhost Research Databases Search Screen - Advanced Search Database - SocINDEX with Full Text
S74	TI tax OR AB tax	Search modes - Boolean/Phrase	Interface - EBSCOhost Research Databases Search Screen - Advanced Search Database - SocINDEX with Full Text
S73	TI program* OR AB program*	Search modes - Boolean/Phrase	Interface - EBSCOhost Research Databases Search Screen - Advanced Search Database - SocINDEX with Full Text
S72	TI strateg* OR AB strateg*	Search modes - Boolean/Phrase	Interface - EBSCOhost Research Databases Search Screen - Advanced Search Database - SocINDEX with Full Text
S71	TI intervent* OR AB intervent*	Search modes - Boolean/Phrase	Interface - EBSCOhost Research Databases Search Screen – Advanced Search Database - SocINDEX with Full Text
S70	TI prevent* OR AB prevent*	Search modes - Boolean/Phrase	Interface - EBSCOhost Research Databases Search Screen - Advanced Search Database - SocINDEX with Full Text
S69	S1 OR S2 OR S3 OR S4 OR S5 OR S6 OR S7 OR S8 OR S9 OR S10 OR S11 OR S12 OR S13 OR S14 OR S15 OR S16 OR S17 OR S18 OR S19 OR S20 OR S21 OR S22 OR S23 OR S24 OR S25 OR S26 OR S27 OR S28 OR S29 OR S30 OR S31 OR S32 OR S33 OR S34 OR S35 OR S36 OR S37 OR S38 OR S39 OR S40 OR S41 OR S42 OR S43 OR S44 OR S45 OR S46 OR S47 OR S48 OR S49 OR S50 OR S51 OR S52 OR S53 OR S54 OR S55 OR S56 OR S57 OR S58 OR S59 OR S60 OR S61 OR S62 OR S63 OR S64 OR S65 OR S66 OR S67 OR S68	Search modes - Boolean/Phrase	Interface - EBSCOhost Research Databases Search Screen - Advanced Search Database - SocINDEX with Full Text

#	Query	Limiters/Expanders	Last Run Via
S68	TI community N5 ties OR AB community N5 ties	Search modes - Boolean/Phrase	Interface - EBSCOhost Research Databases Search Screen - Advanced Search Database - SocINDEX with Full Text
S67	TI community N5 capacity OR AB community N5 capacity	Search modes - Boolean/Phrase	Interface - EBSCOhost Research Databases Search Screen - Advanced Search Database - SocINDEX with Full Text
S66	TI community N5 competenc* OR AB community N5 competenc*	Search modes - Boolean/Phrase	Interface - EBSCOhost Research Databases Search Screen - Advanced Search Database - SocINDEX with Full Text
S65	TI community N5 connect* OR AB community N5 connect*	Search modes - Boolean/Phrase	Interface - EBSCOhost Research Databases Search Screen - Advanced Search Database - SocINDEX with Full Text
S64	TI community N5 skill* OR AB community N5 skill*	Search modes - Boolean/Phrase	Interface - EBSCOhost Research Databases Search Screen - Advanced Search Database - SocINDEX with Full Text
S63	TI community N5 wellbeing OR AB community N5 wellbeing	Search modes - Boolean/Phrase	Interface - EBSCOhost Research Databases Search Screen - Advanced Search Database - SocINDEX with Full Text
S62	TI community N5 well- being OR AB community N5 well-being	Search modes - Boolean/Phrase	Interface - EBSCOhost Research Databases Search Screen - Advanced Search Database - SocINDEX with Full Text
S61	TI community N5 functioning OR AB community N5 functioning	Search modes - Boolean/Phrase	Interface - EBSCOhost Research Databases Search Screen - Advanced Search Database - SocINDEX with Full Text
S60	TI community N5 good OR AB community N5 good	Search modes - Boolean/Phrase	Interface - EBSCOhost Research Databases Search Screen - Advanced Search Database - SocINDEX with Full Text
S59	TI community N5 outcome* OR AB community N5 outcome*	Search modes - Boolean/Phrase	Interface - EBSCOhost Research Databases Search Screen - Advanced Search Database - SocINDEX with Full Text
S58	TI community N5 benefit* OR AB community N5 benefit*	Search modes - Boolean/Phrase	Interface - EBSCOhost Research Databases Search Screen - Advanced Search Database - SocINDEX with Full Text
S57	TI community N5 value* OR AB community N5 value*	Search modes - Boolean/Phrase	Interface - EBSCOhost Research Databases Search Screen - Advanced Search Database - SocINDEX with Full Text
S56	TI community N5 impact OR AB community N5 impact	Search modes - Boolean/Phrase	Interface - EBSCOhost Research Databases Search Screen - Advanced Search Database - SocINDEX with Full Text
S55	TI social N5 cohesion OR AB social N5 cohesion	Search modes - Boolean/Phrase	Interface - EBSCOhost Research Databases Search Screen - Advanced Search Database - SocINDEX with Full Text
S54	TI social N5 inclusion OR AB social N5 inclusion	Search modes - Boolean/Phrase	Interface - EBSCOhost Research Databases Search Screen - Advanced Search Database - SocINDEX with Full Text
S53	TI social N5 ties OR AB social N5 ties	Search modes - Boolean/Phrase	Interface - EBSCOhost Research Databases Search Screen - Advanced Search Database - SocINDEX with Full Text
S52	TI social N5 capacity OR AB social N5 capacity	Search modes - Boolean/Phrase	Interface - EBSCOhost Research Databases Search Screen - Advanced Search Database - SocINDEX with Full Text
S51	TI social N5 competenc* OR AB social N5 competenc*	Search modes - Boolean/Phrase	Interface - EBSCOhost Research Databases Search Screen - Advanced Search Database - SocINDEX with Full Text
S50	TI social N5 connect* OR AB social N5 connect*	Search modes - Boolean/Phrase	Interface - EBSCOhost Research Databases Search Screen - Advanced Search Database - SocINDEX with Full Text

#	Query	Limiters/Expanders	Last Run Via
S49	TI social N5 skill* OR AB social N5 skill*	Search modes - Boolean/Phrase	Interface - EBSCOhost Research Databases Search Screen - Advanced Search Database - SocINDEX with Full Text
S48	TI social N5 wellbeing OR AB social N5 wellbeing	Search modes - Boolean/Phrase	Interface - EBSCOhost Research Databases Search Screen - Advanced Search Database - SocINDEX with Full Text
S47	TI social N5 well-being OR AB social N5 well- being	Search modes - Boolean/Phrase	Interface - EBSCOhost Research Databases Search Screen - Advanced Search Database - SocINDEX with Full Text
S46	TI social N5 functioning OR AB social N5 functioning	Search modes - Boolean/Phrase	Interface - EBSCOhost Research Databases Search Screen - Advanced Search Database - SocINDEX with Full Text
S45	TI social N5 good OR AB social N5 good	Search modes - Boolean/Phrase	Interface - EBSCOhost Research Databases Search Screen - Advanced Search Database - SocINDEX with Full Text
S44	TI social N5 outcome* OR AB social N5 outcome*	Search modes - Boolean/Phrase	Interface - EBSCOhost Research Databases Search Screen - Advanced Search Database - SocINDEX with Full Text
S43	TI social N5 benefit* OR AB social N5 benefit*	Search modes - Boolean/Phrase	Interface - EBSCOhost Research Databases Search Screen - Advanced Search Database - SocINDEX with Full Text
S42	TI social N5 value* OR AB social N5 value*	Search modes - Boolean/Phrase	Interface - EBSCOhost Research Databases Search Screen - Advanced Search Database - SocINDEX with Full Text
S41	TI social N5 impact OR AB social N5 impact	Search modes - Boolean/Phrase	Interface - EBSCOhost Research Databases Search Screen - Advanced Search Database - SocINDEX with Full Text
S40	TI school N5 attendance OR AB school N5 attendance	Search modes - Boolean/Phrase	Interface - EBSCOhost Research Databases Search Screen - Advanced Search Database - SocINDEX with Full Text
S39	TI school N5 readiness OR AB school N5 readiness	Search modes - Boolean/Phrase	Interface - EBSCOhost Research Databases Search Screen - Advanced Search Database - SocINDEX with Full Text
S38	TI academic N5 skill* OR AB academic N5 skill*	Search modes - Boolean/Phrase	Interface - EBSCOhost Research Databases Search Screen - Advanced Search Database - SocINDEX with Full Text
S37	TI academic N5 success OR AB academic N5 success	Search modes - Boolean/Phrase	Interface - EBSCOhost Research Databases Search Screen - Advanced Search Database - SocINDEX with Full Text
S36	TI academic N5 attainment OR AB academic N5 attainment	Search modes - Boolean/Phrase	Interface - EBSCOhost Research Databases Search Screen - Advanced Search Database - SocINDEX with Full Text
S35	TI academic N5 achievement OR AB academic N5 achievement	Search modes - Boolean/Phrase	Interface - EBSCOhost Research Databases Search Screen - Advanced Search Database - SocINDEX with Full Text
S34	TI academic N5 performance OR AB academic N5 performance	Search modes - Boolean/Phrase	Interface - EBSCOhost Research Databases Search Screen - Advanced Search Database - SocINDEX with Full Text
S33	TI academic N5 outcome* OR AB academic N5 outcome*	Search modes - Boolean/Phrase	Interface - EBSCOhost Research Databases Search Screen - Advanced Search Database - SocINDEX with Full Text
S32	TI academic N5 benefit* OR AB academic N5 benefit*	Search modes - Boolean/Phrase	Interface - EBSCOhost Research Databases Search Screen - Advanced Search Database - SocINDEX with Full Text
S31	TI academic N5 value* OR AB academic N5 value*	Search modes - Boolean/Phrase	Interface - EBSCOhost Research Databases Search Screen - Advanced Search Database - SocINDEX with Full Text

#	Query	Limiters/Expanders	Last Run Via
S30	TI academic N5 impact OR AB academic N5 impact	Search modes - Boolean/Phrase	Interface - EBSCOhost Research Databases Search Screen - Advanced Search Database - SocINDEX with Full Text
S29	TI education* N5 skill* OR AB education* N5 skill*	Search modes - Boolean/Phrase	Interface - EBSCOhost Research Databases Search Screen - Advanced Search Database - SocINDEX with Full Text
S28	TI education* N5 success OR AB education* N5 success	Search modes - Boolean/Phrase	Interface - EBSCOhost Research Databases Search Screen - Advanced Search Database - SocINDEX with Full Text
S27	TI education* N5 attainment OR AB education* N5 attainment	Search modes - Boolean/Phrase	Interface - EBSCOhost Research Databases Search Screen - Advanced Search Database - SocINDEX with Full Text
S26	TI education* N5 achievement OR AB education* N5 achievement	Search modes - Boolean/Phrase	Interface - EBSCOhost Research Databases Search Screen - Advanced Search Database - SocINDEX with Full Text
S25	TI education* N5 performance OR AB education* N5 performance	Search modes - Boolean/Phrase	Interface - EBSCOhost Research Databases Search Screen - Advanced Search Database - SocINDEX with Full Text
S24	TI education* N5 outcome* OR AB education* N5 outcome*	Search modes - Boolean/Phrase	Interface - EBSCOhost Research Databases Search Screen - Advanced Search Database - SocINDEX with Full Text
S23	TI education* N5 benefit* OR AB education* N5 benefit*	Search modes - Boolean/Phrase	Interface - EBSCOhost Research Databases Search Screen - Advanced Search Database - SocINDEX with Full Text
S22	TI education* N5 value* OR AB education* N5 value*	Search modes - Boolean/Phrase	Interface - EBSCOhost Research Databases Search Screen - Advanced Search Database - SocINDEX with Full Text
S21	TI education* N5 impact OR AB education* N5 impact	Search modes - Boolean/Phrase	Interface - EBSCOhost Research Databases Search Screen - Advanced Search Database - SocINDEX with Full Text
S20	TI "climate change" N5 outcome* OR AB "climate change" N5 outcome*	Search modes - Boolean/Phrase	Interface - EBSCOhost Research Databases Search Screen - Advanced Search Database - SocINDEX with Full Text
S19	TI "climate change" N5 benefit* OR AB "climate change" N5 benefit*	Search modes - Boolean/Phrase	Interface - EBSCOhost Research Databases Search Screen - Advanced Search Database - SocINDEX with Full Text
S18	TI "climate change" N5 value* OR AB "climate change" N5 value*	Search modes - Boolean/Phrase	Interface - EBSCOhost Research Databases Search Screen - Advanced Search Database - SocINDEX with Full Text
S17	TI "climate change" N5 impact OR AB "climate change" N5 impact	Search modes - Boolean/Phrase	Interface - EBSCOhost Research Databases Search Screen - Advanced Search Database - SocINDEX with Full Text
S16	TI "greenhouse effect" N5 outcome* OR AB "greenhouse effect" N5 outcome*	Search modes - Boolean/Phrase	Interface - EBSCOhost Research Databases Search Screen - Advanced Search Database - SocINDEX with Full Text
S15	TI "greenhouse effect" N5 benefit* OR AB "greenhouse effect" N5 benefit*	Search modes - Boolean/Phrase	Interface - EBSCOhost Research Databases Search Screen - Advanced Search Database - SocINDEX with Full Text
S14	TI "greenhouse effect" N5 value* OR AB "greenhouse effect" N5 value*	Search modes - Boolean/Phrase	Interface - EBSCOhost Research Databases Search Screen - Advanced Search Database - SocINDEX with Full Text
S13	TI "greenhouse effect" N5 impact OR AB "greenhouse effect" N5 impact	Search modes - Boolean/Phrase	Interface - EBSCOhost Research Databases Search Screen - Advanced Search Database - SocINDEX with Full Text
S12	TI "carbon footprint" N5 outcome* OR AB "carbon footprint" N5 outcome*	Search modes - Boolean/Phrase	Interface - EBSCOhost Research Databases Search Screen - Advanced Search Database - SocINDEX with Full Text

#	Query	Limiters/Expanders	Last Run Via
S11	TI "carbon footprint" N5 benefit* OR AB "carbon footprint" N5 benefit*	Search modes - Boolean/Phrase	Interface - EBSCOhost Research Databases Search Screen - Advanced Search Database - SocINDEX with Full Text
S10	TI "carbon footprint" N5 value* OR AB "carbon footprint" N5 value*	Search modes - Boolean/Phrase	Interface - EBSCOhost Research Databases Search Screen - Advanced Search Database - SocINDEX with Full Text
S9	TI "carbon footprint" N5 impact OR AB "carbon footprint" N5 impact	Search modes - Boolean/Phrase	Interface - EBSCOhost Research Databases Search Screen - Advanced Search Database - SocINDEX with Full Text
S8	TI "environment design" N5 outcome* OR AB "environment design" N5 outcome*	Search modes - Boolean/Phrase	Interface - EBSCOhost Research Databases Search Screen - Advanced Search Database - SocINDEX with Full Text
S7	TI "environment design" N5 benefit* OR AB "environment design" N5 benefit*	Search modes - Boolean/Phrase	Interface - EBSCOhost Research Databases Search Screen - Advanced Search Database - SocINDEX with Full Text
S6	TI "environment design" N5 value* OR AB "environment design" N5 value*	Search modes - Boolean/Phrase	Interface - EBSCOhost Research Databases Search Screen - Advanced Search Database - SocINDEX with Full Text
S5	TI "environment design" N5 impact OR AB "environment design" N5 impact	Search modes - Boolean/Phrase	Interface - EBSCOhost Research Databases Search Screen - Advanced Search Database - SocINDEX with Full Text
S4	TI environment* N5 benefit* OR AB environment* N5 benefit*	Search modes - Boolean/Phrase	Interface - EBSCOhost Research Databases Search Screen - Advanced Search Database - SocINDEX with Full Text
S3	TI environment* N5 outcome* OR AB environment* N5 outcome*	Search modes - Boolean/Phrase	Interface - EBSCOhost Research Databases Search Screen - Advanced Search Database - SocINDEX with Full Text
S2	TI environment* N5 value* OR AB environment* N5 value*	Search modes - Boolean/Phrase	Interface - EBSCOhost Research Databases Search Screen - Advanced Search Database - SocINDEX with Full Text
S1	TI environment* N5 impact OR AB environment* N5 impact	Search modes - Boolean/Phrase	Interface - EBSCOhost Research Databases Search Screen - Advanced Search Database - SocINDEX with Full Text

SWiM reporting item

SWiM reporting item	Item description	Page in manuscript where item is reported	Other*
Methods			
1 Grouping studies for synthesis	1a) Provide a description of, and rationale for, the groups used in the synthesis (e.g., groupings of populations, interventions, outcomes, study design)	1-2	
	1b) Detail and provide rationale for any changes made subsequent to the protocol in the groups used in the synthesis	5	
2 Describe the standardised metric and transformation methods used	Describe the standardised metric and transformation methods used Describe the standardised metric for each outcome. Explain why the metric(s) was chosen, and describe any methods used to transform the intervention effects, as reported in the study, to the standardised metric, citing any methodological guidance consulted		Results: 10-14
3 Describe the synthesis methods	Describe and justify the methods used to synthesise the effects for each outcome when it was not possible to undertake a meta-analysis of effect estimates	3-4	
4 Criteria used to prioritise results for summary and synthesis	Where applicable, provide the criteria used, with supporting justification, to select the particular studies, or a particular study, for the main synthesis or to draw conclusions from the synthesis (e.g., based on study design, risk of bias assessments, directness in relation to the review question)	3-4	
5 Investigation of heterogeneity in reported effects	State the method(s) used to examine heterogeneity in reported effects when it was not possible to undertake a meta-analysis of effect estimates and its extensions to investigate heterogeneity	3-4	
6 Certainty of evidence	Describe the methods used to assess certainty of the synthesis findings	3	
7 Data presentation methods	Describe the graphical and tabular methods used to present the effects (e.g., tables, forest plots, harvest plots).<br>Specify key study characteristics (e.g., study design, risk of bias) used to order the studies, in the text and any tables or graphs, clearly referencing the studies included	3-4	
Results			
8 Reporting results	For each comparison and outcome, provide a description of the synthesised findings, and the certainty of the findings. Describe the result in language that is consistent with the question the synthesis addresses, and indicate which studies contribute to the synthesis	10-14	
Discussion			
9 Limitations of the synthesis	Report the limitations of the synthesis methods used and/or the groupings used in the synthesis, and how these affect the conclusions that can be drawn in relation to the original review question	15-16	

Table S1 Summary of study characteristics

Author (year) (n=21)	Setting	Country	Study design & duration	Age group/target population	Sample size	Intervention title/type	Brief description of intervention	Brief description of control/comparator group	Health benefits reported	Co-benefits reported
Bullock 2022[1]	150 elementary, middle and high school	USA	Longitudinal study; 2 years	Aged 2-19 years	1306	Universal free breakfast program	Free school breakfast to reducing financial barriers and improving breakfast participation.	Before policy implementation	Change in number of people who are obese	Education
Caillavet 2016[2]	General population	France	Simulation model; scanner data for 1998–2010	NA	NA	Three food taxation scenarios: Scenario ENV-NUT, ENV and NUT; Food tax	Scenario ENV-NUT: levy on five food groups: beef, cooked meats, animal fats, cheese and prepared mixed meals.	Scenario ENV: taxes all food groups with animal content: beef, other meats, cooked meats, animal fats, cheese, fish and sea foods, dairy products and prepared mixed meals.	Change in energy intake	Environmental
Castiglione 2019[3]	General households	UK	Counterfactual model; Pre-policy (2001–2003); Post-policy (2003–2014)	Mean age (std dev): 51.79 years (16.93 years)	84356	5-a-day policy; Diet	5-a-day campaign at a national level on 25 March 2003 to increase fruit and vegetable intakes towards the World Health Organization (WHO) recommendation of 5 portions (or 400 g) per day.	Pre-policy baseline characteristics	Change in fruit and vegetable intake	Environmental
Colombo 2024	N/A	UK	Modelling; Kantar Fast Moving Consumer Goods (FMCG) Purchase Panel (Take Home) 2017	Adult, aged 19-85		Hypothetical sugar and salt reformulation tax	£3/kg tax on sugar and a £6/kg tax on salt	Current consumption	daily consumption of different food groups and the corresponding energy	Environmental

Author (year) (n=21)	Setting	Country	Study design & duration	Age group/target population	Sample size	Intervention title/type	Brief description of intervention	Brief description of control/comparator group	Health benefits reported	Co-benefits reported
Corcoran 2016[4]	School-based	USA	Longitudinal study; Longitudinal data between 2001/02 and 2011/12. Administrative data between 2006/07 and 2011/12	Elementary and middle grades	284 schools, with an average daily participation of 30,000 students	Breakfast in the Classroom	BIC alters the traditional SBP by offering a free breakfast in class at the start of the school day, rather than in the cafeteria before school hours. The intent is to increase breakfast participation among students who are unable or unwilling to arrive early to school, and to reduce stigma associated with visiting the cafeteria before school for a subsidised meal.	Baseline characteristics of the following groups: never BIC, ever BIC but less than 25%, ever BIC more than 25% but not full and ever BIC but full school.	Change in fruit and vegetable intake	Social
Destaw 2022[5]	School-based	Ethiopia	Single-group repeated measurement/longitudinal study design	Schoolchildren aged 5-19 years old	4 500 schoolchildren	Addis Ababa School Feeding Program (SFP); Multi-component intervention	SFP is a platform for nutritional, health and educational intervention programs such as deworming, micronutrients fortification and supplementation and curricular programmes	Baseline characteristics	Height-for-age, BMI for age z-scores, BMI, and prevalence of stunting and thinness	Education
Gesell 2012[6]	Community-based	USA	Randomised control trial	Latino parent-preschool child dyads	79 parent-preschool child dyads	Salud Con La Familia program; multi-component intervention	All 12 (90 min) weekly group sessions were conducted in Spanish. (e.g., how to choose appropriate portion sizes; how to be active with children; how to monitor screen time).	Do-nothing control group	Absolute BMI (weight [kg]/height [m2])[7]	Social

Author (year) (n=21)	Setting	Country	Study design & duration	Age group/target population	Sample size	Intervention title/type	Brief description of intervention	Brief description of control/comparator group	Health benefits reported	Co-benefits reported
Hernandez 2014[8]	School-based	USA	Cluster randomised, controlled trial; fall of 2006 to spring of 2009	Students from sixth grade to ninth grade	Mean: 1 736 (total), 863 (control), 873 (intervention)	HEALTHY intervention; multi-component intervention	An integrated school-wide program that focused on (1) foods and beverages, (2) physical education, (3) classroom-based behaviour changes and education, and (4) social marketing communication and promotional campaigns.	Schools that did not received HEALTHY interevention.	Change in prevalence of obesity	Education
Izumi 2020[9]	Health center	USA	Single-group pretest–posttest study	English- and Spanish speaking patients	80 patients	CSA program; food subsidy	CSA program memberships enables members to purchase from the farmer for the entire harvest season by paying up front or signing a contract for a share of produce that they pick up weekly at a set location.	Baseline characteristics	Fruit and vegetable intake, frequency of dark green and orange vegetable intake and eating habits	Food security and social
Korn 2018[10]	Community-based	Australia (R&C) and USA (SUS)	Three-phase study design. Phase 1: Survey conceptualisation; Phase 2: Retrospective cohort study with stakeholders; Phase 3: survey modifications and a second prospective reliability assessment.	stakeholders from academia, public schools, foodservice, local health department, community-based organisations.	13 (SUS), 8 (R&C)	Shape Up Somerville (SUS) and Romp & Chomp (R&C); whole-of-community childhood obesity interventions	focus on community capacity-building and developing sustainable changes in areas of policy, sociocultural, and physical environments by using a socio-ecologic framework.	NA	Daily water consumption and daily consumption of fruit and vegetables	Social
Melnyk 2014[11]	School	USA	Quasi-experimental design; 15 weeks	First-year college students; aged 18 to 21 years old	49 students	Freshman 5 to Thrive Course	Freshman 5 to Thrive Course focuses on five core domains of content: (1) cognitive behavioural skills building (CBSB), (2) coping and stress reduction strategies, (3)	The comparison group did not take the Freshman 5 to Thrive Course.	Change in physical activity, nutrition knowledge, intention to	Education

Author (year) (n=21)	Setting	Country	Study design & duration	Age group/target population	Sample size	Intervention title/type	Brief description of intervention	Brief description of control/comparator group	Health benefits reported	Co-benefits reported
							personal development, including goal setting, effective communication, and problem solving, (4) nutrition, and (5) physical activity.		live healthy lifestyle and mental health outcomes	
Melnyk 2013[12]	School	USA	Cluster RCT; 15 weeks	High school students; mean age is 14.74 (SD= 0.73) years old	779 students	COPE (Creating Opportunities for Personal Empowerment) Healthy Lifestyles TEEN (Thinking, Emotions, Exercise, Nutrition) Program	15-session of educational and cognitive-behavioural skills-building program with physical activity component. Each session of COPE contains 15–20 minutes of physical activity and other sessions on diet and eating behaviour, healthy lifestyles, social skills and emotional regulation.	Control program (Healthy Teens) focuses on healthy lifestyle behaviours, BMI, mental health, social skills, and academic performance of high school adolescents	Change in BMI, proportion of overweight, and mental health (anxiety and depression)	Education
Metwally 2020[13]	Primary school	Egypt	Cluster RCT; 5 years	Grade 5 students; mean age is 5 years (SD= 1)	1 789 students	School feeding program; diet	Intervention provides school meal which was a fortified snack (sweet pie) before starting school for students.	Students who had not taken school meal	Weight and height	Education
Monsivais 2015[14]	Home-based	UK	Cross-sectional observational	Adults from European. Age range: 39-79 years old	24293 adults	Dietary Approaches to Stop Hypertension (DASH) diet	DASH diet focuses on plant-based foods, including vegetables and grains, adhering to this diet might also bring about environmental benefits, including lower associated production of greenhouse gases (GHGs).	All participants were assessed for adherence to DASH diet in quintiles.	Energy intake	Environmental
Raine 2013[15]	Community-based	USA	Pre-post intervention; 4 years (Year 2006 – Year 2009)	Four communities in Alberta USA Mean age reported per	29053 participants	"Healthy Alberta Communities" (HAC) project; multiple interventions	Leisure activities, improve diet, promote use of community recreation spaces, improve access to community facilities, support drug, alcohol and tobacco reduction	People who are not a part of the HAC communities	Change in BMI	Social

Author (year) (n=21)	Setting	Country	Study design & duration	Age group/target population	Sample size	Intervention title/type	Brief description of intervention	Brief description of control/comparator group	Health benefits reported	Co-benefits reported
				community (32.3-38.8 years old)		in different communities	program and communication network for organisations to share information.			
Ranjit 2020[16]	Community-based	USA	Cohort-control study; Year 2013-2017 (5 years)	Parents and children. Mean age of child (8.6 years old)	358 parent child dyads	Go! Austin/Vamos! Austin (GAVA) program; multi-component intervention	improve existing built and social environments by increasing access to environmental assets, such as parks and healthy food retail outlets to influence healthy behaviour changes. Four settings of activity were: community food, community PA, school, and early childhood	Control community that was socioeconomically and demographically similar communities drawn from adjacent zip codes to the intervention group	Healthy eating index, eating home-cooked meals	Environmental
Saxe-Custack 2021[17]	School-based	USA	Pre-post survey	Children aged 8-15 years old. Mean age (10.55 years old), sd (1.83)	186 children	Flint Kids Cook program; Education	Farmer's market commercial kitchen teaching students proper cooking techniques and recipes	Baseline characteristics	Change in dietary intake	Education
Schultz 2022[18]	School-based	USA	Pre-post study; 10-week program periods in spring, summer and fall	Grade 3 to 8 Chicago students for fruit and vegetable consumption survey; Grade 9 to 12 students for social-emotional survey	108 students	Gardening-based education program; Education	Students are taught a unique garden-based curriculum and assisted students in growing their school garden. For high school, students participated in after-school sessions and involved in garden internship programs.	Before intervention baseline characteristics	Change in fruit and vegetable intake	Social and education

Author (year) (n=21)	Setting	Country	Study design & duration	Age group/target population	Sample size	Intervention title/type	Brief description of intervention	Brief description of control/comparator group	Health benefits reported	Co-benefits reported
Treu 2017[19]	School-based, homes and a supermarket	USA	Quasi-experimental with three arms. Year 2010 - 2011	Third-grade students. Mean ± SD: 8.7 ± 0.4	1 487 students	Advancing School and Community Engagement Now for Disease Prevention (ASCEND); multi-component	The enhanced intervention (EI) provided daily physical activity in classrooms and a program on making healthful foods, using food labels plus additional components for students and their families such as physical activity kits and family nights. Standard intervention (SI) provided daily physical activity in classrooms and a program on making healthful foods, using food labels.	Control group did not receive any new intervention during the study period but were allowed to continue any programming that they would usually offer, which consisted of physical education classes but no classroom-based programs involving nutrition education or physical activity.	Physical fitness	Education
Wang 2021[20]	National survey	China	Modelling study based on six waves of survey data (1997, 2000, 2004, 2006, 2009 & 2011)	18-75 years old	38 070 participants from 6 waves	Dietary adjustment scenarios (DASs); diet	The study focusses on the following five types of dietary adjustment (A-E). A: Substituting meat with soy products, B: Substituting milk for non-milk animal-derived foods, C: Structural adjust-ment of meat consumption, D: Substituting cereals with tubers, E: Substituting meat with plant-based foods.	Simulation of A-E scenarios	Change in dietary pattern	Environmental

Table S2. Summary of studies reporting educational co-benefits.

Author (year)	Intervention	Co-benefits & outcome measurement instrument (OMIs)	Summary of results on educational co-benefits	
			Performance	Behaviour
Destaw (2022)[5]	**Intervention**: 1-year SFP provided both breakfast and lunch everyday<br>**Comparator:** None	**Co-benefits:** Academic performance and academic behaviour (school attendance)<br>**OMIs:** School records	• Significant increase in the average scores of the schoolchildren over time ($F_{(2,7846)}$ = 108·9, p < 0·0001), indicating improvement in the educational performance	• Average number of school enrolment significantly increased by 4.1% (p<0.01) post intervention.<br>• Absenteeism pre and post intervention declined by 16% (p<0.0001)
Melnyk (2013)[12]	**Interventions:** 15 weeks multi-component intervention delivered by teachers (COPE Program) on cognitive-behavioural skills, physical activity & nutrition education, and socio-emotional management<br>**Comparator:** Healthy Teens program with similar number and length of sessions but focused on common health topics	**Co-benefits:** Academic performance (measured relative academic performance of classmates) [21]<br>**OMIs:** Social Skills Rating Scale (subscale is academic competence) [22]	• Academic performance was significantly higher in the COPE group compared Healthy Teens (MD 1.47 points, 95% CI = 0.05, 2.89).	
Metwally (2020)[13]	**Intervention:** 5 years SFP provided a fortified snack for breakfast<br>**Comparator:** Students who did not receive a free meal from the same school	**Co-benefits:** Academic performance (memory, intelligence, visual-motor performance, auditory vigilance, afternoon attention).<br>**OMIs:** Revised Wechsler Intelligence Scale for children[23]	• Intervention group had significantly higher academic achievements than the control group in the math exam scores (P<0.001)<br>• Intervention groups had higher scores on visual memory, auditory vigilance tests (9.71±2.80 vs. 7.45±3.25; 25.02±3.36 vs. 10.82±8.92, respectively, P<0.001), the afternoon attention and working memory test (8.20±2.21 vs 7.75±3.05) (P<0.001),	

Author (year)	Intervention	Co-benefits & outcome measurement instrument (OMIs)	Summary of results on educational co-benefits	
			Performance	Behaviour
Saxe-Custack (2021)[17]	**Intervention:** 6 weeks Flint Kids Cook program educating students on cooking techniques and recipes<br>**Comparator:** None	**Co-benefits**: Academic behaviour (school functioning which includes attention to class and absenteeism)[24]<br>**OMIs:** Paediatric Quality of Life Inventory Child Self-Report, only on school functioning [25]		• Significant improvement in school functioning score post-intervention (mean = 4.50, p-value= 0.002).
Schultz (2022)[18]	**Intervention:** 10 weeks Gardening-based education program focused on community building, nature, and nutrition<br>**Comparator:** None	**Co-benefits:** Academic behaviour (self-efficacy)<br>**OMIs:** Hello Insight Six Social-Emotional Capacity Areas [26]		• 70% of participants made gains in academic self-efficacy post-intervention.
Bullock (2022)[1]	**Intervention**: 2 years SFP provides free school breakfast to students<br>**Comparator:** Schools with no increase in SFP participation	**Co-benefits**: Academic behaviour (school attendance, unexcused absences, and tardiness)<br>**OMIs:** School records		• There was an average overall increase in school-attendance of 0.3% between the 2012 – 2013 and 2013 – 2014. However, this increase was not significant.<br>• No significant association between change in SFP participation and absence/ attendance and tardiness
Corcoran (2016)[4]	**Intervention**: SFP offered free breakfast at the start of the school day<br>**Comparator:** control schools that never had SFP, or less than 25% of classrooms have SFP, SFP more than 25% of classrooms but not full)	**Co-benefits**: Academic performance (academic test scores) and academic behaviour (attendance rates)<br>**OMIs:** Records from New York Department of Education and Office of School Food	• Statistically significant effect for middle school math achievements, for schools with at least 25% of classrooms received SFP, a positive 0.007 SD per 100 days ($p < 0.05$).	• Differences in attendance from baseline were small in magnitude and statistically insignificant.
Hernandez (2014)[8]	**Intervention:** 3 years HEALTHY intervention, a multi-component intervention targeted nutrition (e.g. education) physical activity (e.g. dance, football), and the social environment (e.g. cooperative/adventure games)[27]	**Co-benefits:** Academic performance (academic test scores) and academic behaviour (school attendance rates)<br>**OMIs:** School records	• There were no differences between intervention and control schools in educational performance for mathematics (p = 0.78) or reading (p = .63)	• There was a significant decrease in annual rates of disciplinary action from sixth to eighth grades in both the study cohort (p = 0.0394) and school-wide population (p = 0.0201).

Author (year)	Intervention	Co-benefits & outcome measurement instrument (OMIs)	Summary of results on educational co-benefits	
			Performance	Behaviour
	**Comparator:** Control schools			• No difference in school attendance rates (p = 0.582) and disciplinary action (p=0.8671).
Melnyk (2014)[11]	**Intervention**: 15 weeks Freshman 5 to Thrive/ COPE, a multi-component intervention with nutrition, physical activity, and coping strategies<br>**Comparator:** Freshmen who did not take Freshman 5 to Thrive course	**Co-benefits:** Academic behaviour (college retention)<br>**OMIs:** University data		• Retention in intervention group was 9.6% higher compared to students not enrolled in intervention. No further details reported.
Treu (2017)[28]	**Intervention**: Two multi-component interventions, Standard intervention (SI) (focused on physical activity, healthy food, education) and enhanced intervention (EI) with additional components (such as night educational sessions and take-home educational kits) over 2 terms.<br>**Comparator:** usual practice control students	**Co-benefits**: Academic performance (reading comprehension, reading fluency, problem solving, computation performance)<br>**OMIs**: MAZE (reading test), R-CBM (Reading Curriculum-Based Measurement), M-CAP (Mathematics Concepts and Applications) and M-CBM (Mathematics Curriculum-Based Measurement) [29]	• MAZE scores increased significantly in the SI group and EI group compared to control. Between-group differences p-values SI vs control = <0.001, EI vs control = 0.03, EI vs SI = <0.001).<br>• M-CBM scores significantly less favourable for SI and EI compared to control.<br>• M-CAP and, R-CBM score also favouring control but statistically non-significant.	• No significant difference between groups for classroom behaviour

Table notes: OMIs = outcome measurement instruments; SFP = School feeding program; COPE = Creating Opportunities for Personal Empowerment;

Healthy Lifestyles TEEN Program = Thinking, Emotions, Exercise, Nutrition

Table S3. Summary of studies reporting social co-benefits.

Author (year)	Intervention	Co-benefits & outcome measurement instrument (OMIs)	Summary of results
Gesell (2012)[6]	**Intervention**: a culturally tailored multi-component intervention aimed at improving family nutritional habits, increasing physical activity, and decreasing screen time<br>**Comparator:** brief school readiness program	**Co-benefits**: Social connection<br>**OMIs**: Social Network Structure measured via repeat surveys	• Significant increase in social ties after intervention by a MD 2.52 (p =0.006) compared to control.<br>• Significant increase in degree of person's ties with others in intervention compared to control by 0.16 (p=0.011).<br>• Significant increase in all potential social ties in network after intervention by 8.54% (p=0.014) comparing to control.<br>• The significant increased tendency for parents to form new friendship ties with other parents whose child's BMI z-score is similar to their own child, (p <0.05).
Raine (2013)[15]	**Intervention:** Healthy Alberta Communities multi-component intervention aimed at improving food accessibility, social inclusion, and access to healthy food<br>**Comparator:** None	**Co-benefits:** Sense of belonging<br>**OMIs:** Computer Assisted Telephone Interview questionnaire to compare changes in self-report health, social conditions and behavioural indicators.	• Significant increase in sense of belonging (p<0.001) to community among intervention group from baseline to post-intervention.
Saxe-Custack (2021)[17]	**Intervention:** Flint Kids Cook program educating students on cooking techniques and recipes<br>**Comparator:** None	**Co-benefits**: Social functioning (social interactions with other children)[24]<br>**OMIs:** Paediatric Quality of Life Inventory Child Self-Report	• Significant increase in social functioning by 3.18 (p = 0.037) and emotional functioning by 5.31 (p = 0·002).<br>• Significant correlation between cooking self-efficacy and change in psychosocial health summary score (r = –0·27, P = 0·002).

Author (year)	Intervention	Co-benefits & outcome measurement instrument (OMIs)	Summary of results
Schultz (2022)[18]	**Intervention:** Gardening-based education program<br>**Comparator:** None	**Co-benefits:** Social-emotional capacities such as positive identity, contribution and social skills<br>**OMIs:** Hello Insight contribution and social skills[26]	There were statistically significant gains in all five social and emotional capacities post intervention:<br>• 70% made gains in Positive Identity<br>• 74% made gains in Self-Management<br>• 67% made gains in Contribution<br>• 77% made gains in Social Skills.
Melnyk (2013)	**Interventions**: 15 weeks multi-component intervention delivered by teachers (COPE Program) on cognitive-behavioural skills, physical activity & nutrition education, and socio-emotional management<br>**Comparator**: Healthy Teens program with similar number and length of sessions but focused on common health topics	**Co-benefits:** cooperation, assertation<br>**OMIs:** Social Skills Rating Scale[22]	There was a significant difference post-intervention in cooperation scale (0.904 (95% CI 0.129, 1.68) and assertion scale (2.89 (95% CI 2.00, 3.77), favouring COPE program over Healthy Teens.
Korn (2018)[10]	**Intervention**: Shape-Up Somerville (SUP) - community-based environmental change intervention in elementary schools, and Romp&Chomp (R&C) - capacity-building and environmental intervention targeting children from birth to five years<br>**Comparator:** None	**Co-benefits**: Social networks, engagement and dialogue-specific outcomes (dialogue and mutual learning, flexibility in their willingness to compromise or adapt, influence and power on events, other people's thinking and their behaviour, leadership and stewardship, and trust in others)<br>**OMI**: Social network survey designed to enable quantification of social networks[30 31], and Community-Based Participatory Research Model to assess engagement of stakeholders[32-34].	Baseline to follow-up:<br>• Non-significant increase in engagement for both SUS and R&C.<br>• R&C showed no significant change in dialogue-specific outcomes.<br>• SUS had a significant increase in the influence & power sub-domain of dialogue-specific outcomes (mean score =0.31, 95% CI =0.02, 0.59, p-value = <0.05).<br>• The other domains showed no significant change for SUS.

Author (year)	Intervention	Co-benefits & outcome measurement instrument (OMIs)	Summary of results
Izumi (2020)[9]	**Intervention:** Community-Supported Agriculture (CSA)<br>**Comparator:** None	**Co-benefits:** Socialising and taking part in social activities<br>**OMIs:** Duke Health Profile[35]	• No significant increase was reported for socialising and taking part in social activities.
Ranjit (2020)[16]	**Intervention:** Go! Austin/Vamos! Austin program is a multi-component intervention aimed at improving the built environment, physical activity and nutrition.<br>**Comparator:** Unexposed control group from a different community	**Co-benefits:** Perception of community facilities<br>**OMIs:** Survey on the perceptions of availability and quality of PA facilities and PA programs in the community.	• Changes in perception of condition of neighbourhood facilities were not significant.<br>• Significantly more areas were considered not safe for teens or adults to bike/walk in neighbourhood ($p = 0.0005$).

Table notes: SUS= Shape Up Somerville, R&C= Romp & Chomp; COPE = Creating Opportunities for Personal Empowerment, IG= intervention group, CG= control group

Table 4 Summary of studies reporting environmental co-benefits

Author (year)	Intervention	Co-benefits & outcome measurement instrument (OMIs)	Summary of results
Caillavet (2016)[2]	**Intervention**: simulation of two tax (20%) scenarios<br>1) Animal-based foods that are high in GHGE (ENV-NUT)<br>2) All animal-based foods (ENV).<br>**Comparator:** Current diet based on food consumption data 1998-2010	**Co-benefits**: CO_2, SO_2 and N emissions<br>**OMIs**: Life Cycle Analysis [36] to identify CO_2, SO_2 and N emissions of different consumption patterns	When compared to baseline emission:<br>• Taxation scenario 1 (ENV) had reduction in CO_2 by 7.51%; ENV-NUT, resulted in CO_2 reduction of 6.96%.<br>• ENV had reduction of SO_2 by 14.52% (mean 37.97) and ENV-NUT resulted in a reduction in SO_2 of 13.21%.<br>• ENV had a reduction in N (8.36%); ENV-NUT, resulted in reduction in N of 6.62%.
Castiglione (2019)[3]	**Intervention**: The "5-a-day" programme aimed at increasing fruit and vegetable intake<br>**Comparator:** Current diet	**Co-benefits:** GHGE quantified as CO_2 equivalents in kg/person/month.<br>**OMIs:**<br>Expenditure and Food Survey (EFS) and the Living Cost Survey (LCS) [37] to identify purchases and household waste to identify GHGEs.	• Increase in fruit and vegetable intake resulted in a reduction of 3.3 kg CO_2e per adult per month. No p-value reported.<br>• The study estimates a significant decrease in purchases for 15 out of 17 groups, and the net outcome is a reduction of GHGEs estimated at −3.66 kg CO_2e per adult per month.
Colombo (2024)	**Intervention:** £3/kg tax on sugar and a £6/kg tax on salt<br>**Comparator:** Current consumption without tax	**Co-benefits**: CO_2, SO_2 and N emissions<br>**OMIs:** meta-analysis of food product Life Cycle Assessments from literature was used to estimate the diet-related GHGE for each product in 8 food groups: biscuit, crackers, bread, breakfast cereals, confectionery, desserts, snacks, spreads.	Environmental impacts across eight food groups when comparing consumption of foods high in salt/ sugar with substitutes lower in salt/sugar:<br>• Daily change in g of CO_2e for all food categories: -2.7 (Mt)<br>• Daily change in g of PO_4 e for all food categories: -11 697 (tonnes)<br>• Daily change in g of SO_2e: -13 769 tonnes.

Author (year)	Intervention	Co-benefits & outcome measurement instrument (OMIs)	Summary of results
Monsivais (2015)[14]	**Intervention**: Dietary Approaches to Stop Hypertension<br>**Comparator:** None	**Co-benefits:** GHGE CO_2 equivalent in kg/day<br>**OMIs:**<br>Based on a food-frequency questionnaire [38] food and nutrition database, GHGEs for the 289 food codes were constructed	• Diets with highest adherence to Dietary Approaches to Stop Hypertension reported significant reduction in CO_2 equivalents, kg/d compared to lowest diet adherence by 16.5% (pairwise test comparing highest and lowest quintile p-value = <0.0001).<br>• Red and processed meats had the strongest association, in which the most accordant diets (Q5) had an associated GHG impact that was 4.3 kg CO_2eq/d (49.5%) lower than the GHG impact associated with Q1 diets.
Wang (2021)[20]	**Intervention**: Dietary adjustment scenarios to reduce the simultaneous risks of obesity/overweight and CF.<br>Scenario A: Substituting meat with soy products and white meat<br>Scenario B: Substituting of red meat with milk<br>Scenario C: Structural adjustment of meat consumption<br>Scenario D: Substituting cereals with tubers<br>Scenario E: Substituting meat with plant-based foods.<br>**Comparator:** None	**Co-benefits:** modelled GHGEs including CO_2, CH_4, and N_2O in kg per person/year<br>**OMIs**: China Health and Nutrition Survey [39] data to extract food names matched to China Food composition tables [40]. CF of the food production system was calculated using the Intergovernmental Panel on Climate Change method, multiplying the emission factors by the activity data.	• Overall, CF changes for substitution of beef with vegetables produced most benefit on CF reduction by 2.65 kg CO_2e<br>• Scenario A: Substitution of red meat with milk, soy products, and white meat produces a 9.1 Mt, 6.5 Mt, and 3.4 Mt CO_2e annual CF reduction in China under an assumption that half of the population adopt the dietary substitution twice a week.<br>• Scenario B: A substitution of red meat with milk have CF reduced by 1.85 to 2.53 kg CO_2e per person per year.<br>• Scenario C: In the scenario of substituting beef with white meat, the CF was reduced by 2.6 kg CO_2e. Substituting mutton with white meat, reduced CF by 1.9 kg CO_2e. In substituting mutton with beef, the CF increased by 0.68 kg CO_2e annually.<br>• Scenario D: On the scenario substituting cereals with tubers had limited effects except for a slight reduction in CF of 0.01 kg CO_2e annually.<br>• Scenario E: On the scenario of substituting red meat with vegetables or cereals, the largest reduction in CF was 2.61 kg CO_2e; substituting white meat with plant-based foods cause a reduction of 0.08 kg CO_2e in C annually.

Table notes: GHGEs= greenhouse gas emissions, CF= carbon footprint, Mt= mega Tones, CO_2= carbon dioxide, SO_2= sulphur dioxide, PO4eq = phosphate equivalents (eutrophication potential), N= nitrogen dioxide, kg= kilogram

Reference

1. Bullock SL, Dawson-McClure S, Truesdale KP, et al. Associations between a Universal Free Breakfast Policy and School Breakfast Program Participation, School Attendance, and Weight Status: A District-Wide Analysis. *International journal of environmental research and public health* 2022;19(7) doi: 10.3390/ijerph19073749
2. Caillavet F, Fadhuile A, Nichèle V. Taxing animal-based foods for sustainability: environmental, nutritional and social perspectives in France. *European Review of Agricultural Economics* 2016;43(4):537-60. doi: 10.1093/erae/jbv041https://erae.oxfordjournals.org/content/43/4/537.abstract
3. Castiglione C, Mazzocchi M. Ten years of five-a-day policy in the UK: Nutritional outcomes and environmental effects. *Ecological Economics* 2019;157:185-94. doi: 10.1016/j.ecolecon.2018.11.016
4. Corcoran SP, Elbel B, Schwartz AE. The Effect of Breakfast in the Classroom on Obesity and Academic Performance: Evidence from New York City. *Journal of policy analysis and management : [the journal of the Association for Public Policy Analysis and Management]* 2016;35(3):509-32. doi: 10.1002/pam.21909
5. Destaw Z, Wencheko E, Kidane S, et al. Impact of school meals on educational outcomes in Addis Ababa, Ethiopia. *Public health nutrition* 2022;25(9):2614-24. doi: 10.1017/S1368980022000799
6. Gesell SB, Bess KD, Barkin SL. Understanding the Social Networks That Form within the Context of an Obesity Prevention Intervention. *Journal of obesity* 2012;2012:749832. doi: 10.1155/2012/749832
7. Barkin SL, Gesell SB, Po'e EK, et al. Culturally tailored, family-centered, behavioral obesity intervention for Latino-American preschool-aged children. *Pediatrics* 2012;130(3):445-56. doi: 10.1542/peds.2011-3762 [published Online First: 2012/08/08]
8. Hernandez AE, Marcus MD, Hirst K, et al. Impact of implementation and conduct of the HEALTHY primary prevention trial on student performance. *American journal of health promotion : AJHP* 2014;29(1):55-58. doi: 10.4278/ajhp.130131-ARB-53
9. Izumi BT, Martin A, Garvin T, et al. CSA Partnerships for Health: outcome evaluation results from a subsidized community-supported agriculture program to connect safety-net clinic patients with farms to improve dietary behaviors, food security, and overall health. *Translational behavioral medicine* 2020;10(6):1277-85. doi: 10.1093/tbm/ibaa041
10. Korn AR, Hennessy E, Hammond RA, et al. Development and testing of a novel survey to assess Stakeholder-driven Community Diffusion of childhood obesity prevention efforts. *BMC public health* 2018;18(1):681. doi: 10.1186/s12889-018-5588-1
11. Melnyk B, Kelly S, Jacobson D, et al. Improving physical activity, mental health outcomes, and academic retention in college students with Freshman 5 to Thrive: COPE/Healthy Lifestyles. *Journal of the American Association of Nurse Practitioners* 2014;26(6):314-22. doi: 10.1002/2327-6924.12037
12. Melnyk BM, Jacobson D, Kelly S, et al. Promoting healthy lifestyles in high school adolescents: a randomized controlled trial. *American journal of preventive medicine* 2013;45(4):407-15. doi: 10.1016/j.amepre.2013.05.013
13. Metwally AM, El-Sonbaty MM, El Etreby LA, et al. Impact of National Egyptian school feeding program on growth, development, and school achievement of school children. *World journal of pediatrics : WJP* 2020;16(4):393-400. doi: 10.1007/s12519-020-00342-8
14. Monsivais P, Scarborough P, Lloyd T, et al. Greater accordance with the Dietary Approaches to Stop Hypertension dietary pattern is associated with lower diet-related greenhouse gas production but higher dietary costs in the United Kingdom. *The American journal of clinical nutrition* 2015;102(1):138-45. doi: 10.3945/ajcn.114.090639

15. Raine K, Plotnikoff R, Schopflocher D, et al. Healthy Alberta communities impact of a three-year community-based obesity and chronic disease prevention intervention. *Obesity Research and Clinical Practice* 2013;7:e106. doi: 10.1016/j.orcp.2013.12.690
16. Ranjit N, Nielsen A, Akhavan N, et al. Outcomes of a community-wide health intervention in a low-income, primarily hispanic community: the Go! Austin/Vamos! Austin (Gava) initiative. *Health Promotion Practice* 2020;23(1):185-94. doi: 10.1177/1524839920961365https://journals.sagepub.com/doi/full/10.1177/1524839920961365
17. Saxe-Custack A, LaChance J, Hanna-Attisha M, Dawson C. Flint Kids Cook : positive influence of a farmers' market cooking and nutrition programme on health-related quality of life of US children in a low-income, urban community. *Public health nutrition* 2021;24(6):1492-500. doi: 10.1017/S136898002000395X
18. Schultz C, Rosen AE. School Gardens' Impact on Students' Health Outcomes in Low-Income Midwest Schools. *The Journal of school nursing : the official publication of the National Association of School Nurses* 2022;38(5):486-93. doi: 10.1177/10598405221080970
19. Treu JA, Doughty K, Reynolds JS, et al. Advancing School and Community Engagement Now for Disease Prevention (ASCEND). *American journal of health promotion : AJHP* 2017;31(2):143-52. doi: 10.4278/ajhp.140820-QUAN-413
20. Wang L, Cui S, Hu Y, et al. The co-benefits for food carbon footprint and overweight and obesity from dietary adjustments in China. *Journal of Cleaner Production* 2021;289 doi: 10.1016/j.jclepro.2020.125675https://www.sciencedirect.com/science/article/abs/pii/S0959652620357218
21. Doll BJ, Meikamp L-FJJW. Social Skills Improvement System Rating Scales. SSIS Rating Scales, 2010.
22. Gresham FM, & Elliot, S. N. Social Skills Rating System. *APA PsycTests* 1990 doi: https://psycnet.apa.org/doi/10.1037/t10269-000
23. Grizzle R. Wechsler Intelligence Scale for Children, Fourth Edition. In: Goldstein S, Naglieri JA, eds. Encyclopedia of Child Behavior and Development. Boston, MA: Springer US 2011:1553-55.
24. Lauren K, Brenda B, Tanuja C, et al. Effect of fingolimod on health-related quality of life in paediatric patients with multiple sclerosis: results from the phase 3 PARADIGMS Study. *BMJ Neurology Open* 2022;4(1):e000215. doi: 10.1136/bmjno-2021-000215
25. Anna Freud National Centre for Children and Families. Paediatric Quality of Life (PedsQL) nd [Available from: https://www.corc.uk.net/outcome-experience-measures/paediatric-quality-of-life-pedsql/.
26. Hi. Hello Insight nd [Available from: https://helloinsight.org/.
27. McMurray RG, Bassin S, Jago R, et al. Rationale, design and methods of the HEALTHY study physical education intervention component. *Int J Obes (Lond)* 2009;33 Suppl 4(Suppl 4):S37-43. doi: 10.1038/ijo.2009.115 [published Online First: 2009/07/23]
28. Treu JA, Doughty K, Reynolds JS, et al. Advancing School and Community Engagement Now for Disease Prevention (ASCEND): A Quasi-experimental Trial of School-Based Interventions to Prevent Childhood Obesity. *American journal of health promotion : AJHP* 2017;31(2):143-52. doi: 10.4278/ajhp.140820-QUAN-413
29. Pearson. aimsweb PLUS nd [Available from: https://www.pearsonassessments.com/store/usassessments/en/Store/Professional-Assessments/Academic-Learning/Brief/aimswebPlus/p/100000519.html?tab=overview.
30. McGlashan J, Nichols M, Korn A, et al. Social network analysis of stakeholder networks from two community-based obesity prevention interventions. *PLoS One* 2018; 13(4). http://europepmc.org/abstract/MED/29702660

https://doi.org/10.1371/journal.pone.0196211

https://europepmc.org/articles/PMC5922530

https://europepmc.org/articles/PMC5922530?pdf=render

https://journals.plos.org/plosone/article/file?id=10.1371/journal.pone.0196211&type=printable (accessed 2018).

31. Marsden PV. Survey Methods for Network Data. In: John S, Peter JC, eds. The Sage Handbook of social Network Analysis. London: Sage Publications 2011:370-88.
32. Sandoval JA, Lucero J, Oetzel J, et al. Process and outcome constructs for evaluating community-based participatory research projects: a matrix of existing measures. *Health Educ Res* 2012;27(4):680-90. doi: 10.1093/her/cyr087 [published Online First: 2011/09/24]
33. Wallerstein N, Duran B. Community-Based Participatory Research Contributions to Intervention Research: The Intersection of Science and Practice to Improve Health Equity. *American Journal of Public Health* 2010;100(S1):S40-S46. doi: 10.2105/AJPH.2009.184036
34. Wallerstein N, Oetzel J, Duran B, et al. What predicts outcomes in CBPR?2008:371-92.
35. Parkerson GR, Jr., Broadhead WE, Tse CK. The Duke Health Profile. A 17-item measure of health and dysfunction. *Med Care* 1990;28(11):1056-72. doi: 10.1097/00005650-199011000-00007 [published Online First: 1990/11/01]
36. Goedkoop M, Heijungs R, Huijbregts M, et al. ReCiPE 2008: A life cycle impact assessment method which comprises harmonised category indicators at the midpoint and the endpoint level. 2008
37. Office for National Statistics DfE, Food and Rural Affairs,. Living Costs and Food Survey, 2001–2014: UK Data Service, 2017.
38. Welch AA, Luben R, Khaw KT, Bingham SA. The CAFE computer program for nutritional analysis of the EPIC-Norfolk food frequency questionnaire and identification of extreme nutrient values. *Journal of Human Nutrition and Dietetics* 2005;18(2):99-116. doi: https://doi.org/10.1111/j.1365-277X.2005.00593.x
39. China Health and Nutrition Survey. 2015 [Available from: https://www.cpc.unc.edu/projects/china.
40. Yang YX, Wang GY, Pan XC. China food composition. *Peking University Medical Press: Beijing, China* 2009;42:795-99.
